# Cell spinpods are a simple inexpensive suspension culture device to deliver fluid shear stress to renal proximal tubular cells

**DOI:** 10.1038/s41598-021-00304-8

**Published:** 2021-10-29

**Authors:** Timothy G. Hammond, Corey Nislow, Ivan C. Christov, Vecihi Batuman, Pranay P. Nagrani, Marjan Barazandeh, Rohit Upadhyay, Guri Giaever, Patricia L. Allen, Michael Armbruster, Allen Raymond, Holly H. Birdsall

**Affiliations:** 1 Cell Spinpod LLC, Chapel Hill, NC 27516 USA; 2 Nephrology Division, Department of Internal Medicine, Duke University School of Medicine, Durham, NC 27705 USA; 3 Space Policy Institute, Elliott School of International Affairs, George Washington University, Washington, DC 20052 USA; 4 Nephrology Section, John W. Deming Department of Medicine, Tulane University School of Medicine, New Orleans, LA 70112 USA; 5 Nephrology Section, Medicine Service Line, Durham VA Health Care System, Building 15, Room 210, 508 Fulton Street, Durham, NC 27705 USA; 6 Faculty of Pharmaceutical Sciences, The University of British Columbia, Vancouver, BC V6T 1Z3 Canada; 7 School of Mechanical Engineering, Purdue University, West Lafayette, IN 47907 USA; 8 Incept 3D, San Diego, CA 92121 USA; 9 Rite Tech Industries Inc., Trinity, FL 34655 USA; 10 Departments of Otorhinolaryngology, Immunology, and Psychiatry, Baylor College of Medicine, Houston, TX 77030 USA; 11 Otolaryngology Section, Surgery Service Line, Durham VA Health Care System, Building 15, Room 210, 508 Fulton Street, Durham, NC 27705 USA

**Keywords:** Translational research, Biological models

## Abstract

Rotating forms of suspension culture allow cells to aggregate into spheroids, prevent the de-differentiating influence of 2D culture, and, perhaps most importantly of all, provide physiologically relevant, in vivo levels of shear stress. Rotating suspension culture technology has not been widely implemented, in large part because the vessels are prohibitively expensive, labor-intensive to use, and are difficult to scale for industrial applications. Our solution addresses each of these challenges in a new vessel called a cell spinpod. These small 3.5 mL capacity vessels are constructed from injection-molded thermoplastic polymer components. They contain self-sealing axial silicone rubber ports, and fluoropolymer, breathable membranes. Here we report the two-fluid modeling of the flow and stresses in cell spinpods. Cell spinpods were used to demonstrate the effect of fluid shear stress on renal cell gene expression and cellular functions, particularly membrane and xenobiotic transporters, mitochondrial function, and myeloma light chain, cisplatin and doxorubicin, toxicity. During exposure to myeloma immunoglobulin light chains, rotation increased release of clinically validated nephrotoxicity cytokine markers in a toxin-specific pattern. Addition of cisplatin or doxorubicin nephrotoxins reversed the enhanced glucose and albumin uptake induced by fluid shear stress in rotating cell spinpod cultures. Cell spinpods are a simple, inexpensive, easily automated culture device that enhances cellular functions for in vitro studies of nephrotoxicity.

## Introduction

Cells throughout the body are exposed to flow shear stress as fluids stream past their membranes^1,2^. In the kidney, fluid from the blood is filtered by the glomerulus, and this ultrafiltrate flows past proximal tubule cells (PTC) that are responsible for reabsorbing water, sodium, glucose, amino acids, and diverse hormones and proteins. This is not a languid process—a healthy kidney generates over 100 mL of ultrafiltrate per minute and the PTC are responsible for reabsorbing 70% of this volume. During these processes, cells of the proximal tubule experience fluid shear stress. Calculations of the actual fluid shear stress in vivo is complicated by the varying dimensions of the tubules and varying composition of the ultrafiltrate as it moves down the tubule, but it is estimated that PTC are exposed to shear stress in the range of 0.04–0.50 dynes/cm^2^^1,3–5^.

Fluid shear stress has an important role in maintaining the differentiation of PTC^2,6^. Exposure to fluid shear stress in vitro increases PTC transport of proteins^7–12^, expression of microvilli^10^, and formation of tight junctions, with increased transepithelial electrical resistance^13–19^. Accordingly, in order to serve as meaningful and representative models of living kidneys, cultured PTC in vitro must be exposed to fluid shear stress in a quantifiable manner^2,20^.

Generating fluid shear stress in vitro presents a variety of challenges (reviewed in^2^). Several technologies are available including orbital shakers, parallel plates, microfluidics with peristaltic pumps, and perfused hollow fibers^8,13,21–24^. Roller bottles, paddle stirrers, and shakers are inexpensive options that are quite suitable for microorganisms with rigid cell walls such as fungi, bacteria, and algae that can tolerate the high shear levels induced by turbulent flow and are relatively resistant to injury from impact against the vessel walls and paddles. However, mammalian cells require much gentler treatment to avoid cellular damage and to accurately mimic the shear levels they experience in vivo^5,25^. Microfluidic and parallel plate models can deliver low levels of fluid shear stress but are rarely practical for high throughput analysis due to high cost, complexity, and/or large vessel volumes^2^.

Rotating suspension cultures, where cells float in a liquid milieu, have significant advantages for delivering physiologic levels of flow shear stress. Suspension culture technology has been modeled, validated experimentally, and partially standardized for routine use^6,26,27^. In a rotating suspension culture with no headspace (i.e. when the chamber is completely full of media), the cells and medium rotate as a cohesive mass in laminar flow with no turbulence^1,28^. The rotating wall vessel spins around a horizontal axis and the cells move in an annulus around the axis of rotation (Supplemental Video S1). As the cells tend to sediment under the influence of gravity, the rotation of the vessel, brings them back up into suspension. Adherence-dependent cells can still be grown in rotating suspension culture vessels after attachment to microcarrier beads.

Rotating suspension cultures not only avoid the limitations of 2D cultures, but also expose free cells, cells on the surface of microcarrier beads, or cells in spheroids to continuous and uniform levels of fluid shear stress. In a rotating suspension culture with zero headspace, cells are exposed to ~ 0.04 to 0.50 dynes/cm^2^ of shear stress^1,6,29–32^. It is important to note that the rotation speed does not affect shear stress, but rather determines the diameter of the annulus through which the cells migrate. Hence, rotating suspension culture delivers the same shear on the same size and density cells in the same media every time regardless of rotation speed. This gives the investigator broad parameters for rotation speed and facilitates delivery of reproducible shear stress.

Despite its advantages, rotating suspension culture has found limited application due to limitations of the currently available hardware^2^. For example, re-usable vessels have multiple components, each requiring sterilization by autoclaving at different temperatures, as well as requiring complex manual assembly in a sterile environment. During use, the vessels attach to spindle rotators that spin with great precision. However, the rotators are relatively expensive and only accommodate a few vessels at one time, constraining the design of experiments in which large numbers of replicates are desirable^2^. In the most common applications, rotating suspension cultures are used to generate a large batch of tissue spheroids that are then transferred to other (static) systems for experimentation; in this case the advantages of suspension culture are lost at the analysis step.

There is a compelling need for small, affordable, simple-to-use suspension culture devices for studies with large numbers of replicates^2,6^. We report the development and utility of an inexpensive, simple to use, rotating suspension culture device, called a cell spinpod. Fluid shears in these injection-molded cell spinpods are illustrated with two-fluid modeling of flow and stresses. To demonstrate their utility, we used cell spinpods to deliver fluid shear stress to immortalized renal proximal tubule cells growing as a confluent monolayer on the surface of collagen carrier beads. The rotation of the cell spinpod keeps the beads in suspension and exposes the cells to low levels of fluid shear stress. With this model, we have demonstrated the effect of fluid shear stress on renal cell gene expression and cellular functions, particularly cell transporters, and the consequences of myeloma light chain, cisplatin, and doxorubicin toxicity. These studies demonstrate that immortalized renal cells exposed to fluid shear stress in cell spinpods can be a useful target for in vitro nephrotoxicity assays.

## Materials and methods

### Cell spinpods

**Figure 1:**
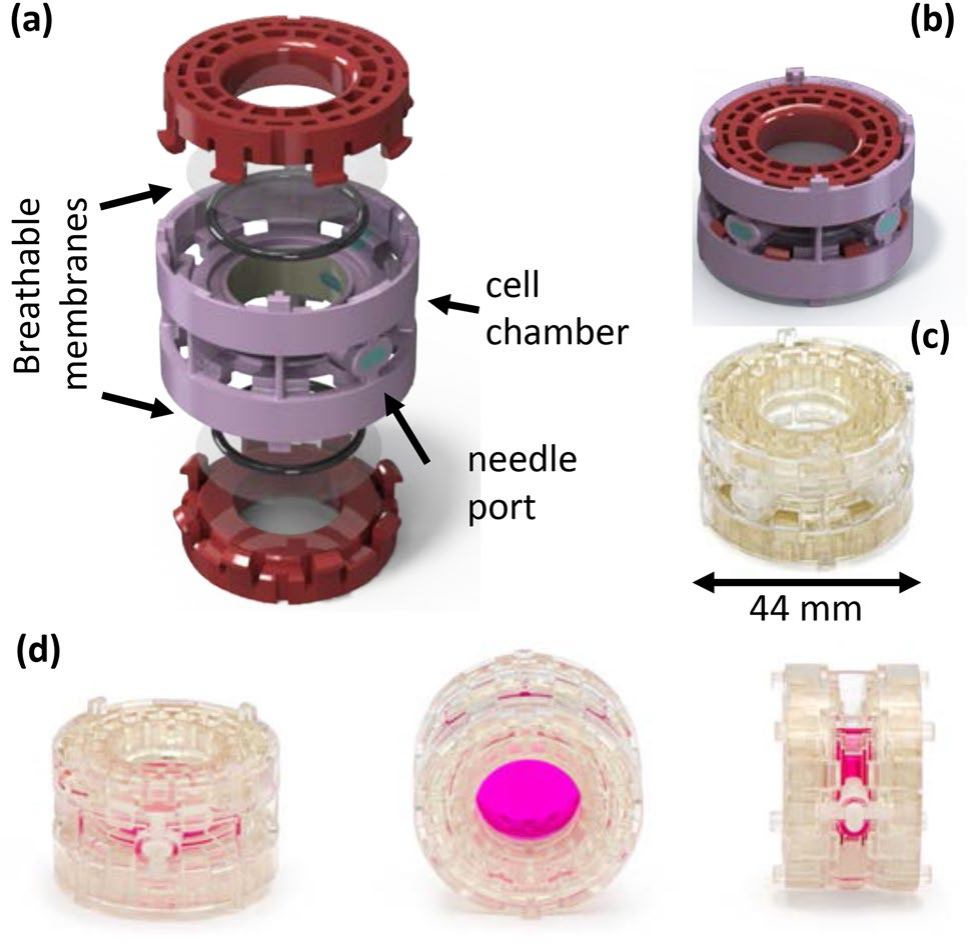
Components of the cell spinpod. Components of the cell spinpod. Panels (**a**) and (**b**) are CAD renderings prepared by author AR, with SolidWorks 2020 Professional Service Pack 4 (https://www.solidworks.com/lp/just-cad). The cell/media chamber, in lilac, is sandwiched between two breathable membranes that are sealed on two sides with silicone rubber O-rings and held in place with retaining caps (shown in red) with eight snap features on each. There are two surface-bonded self-sealing silicone rubber needle ports (shown in turquoise) incorporated through the side walls of the cell chamber, for loading cells/media and for bleeding the air during loading. Panel (**c**) shows an actual empty cell spinpod and Panel (**d**) shows three views of a cell spinpod filled with pink media. Photographs in panels (**c**) and (**d**) were taken by author HB.

**Figure 2:**
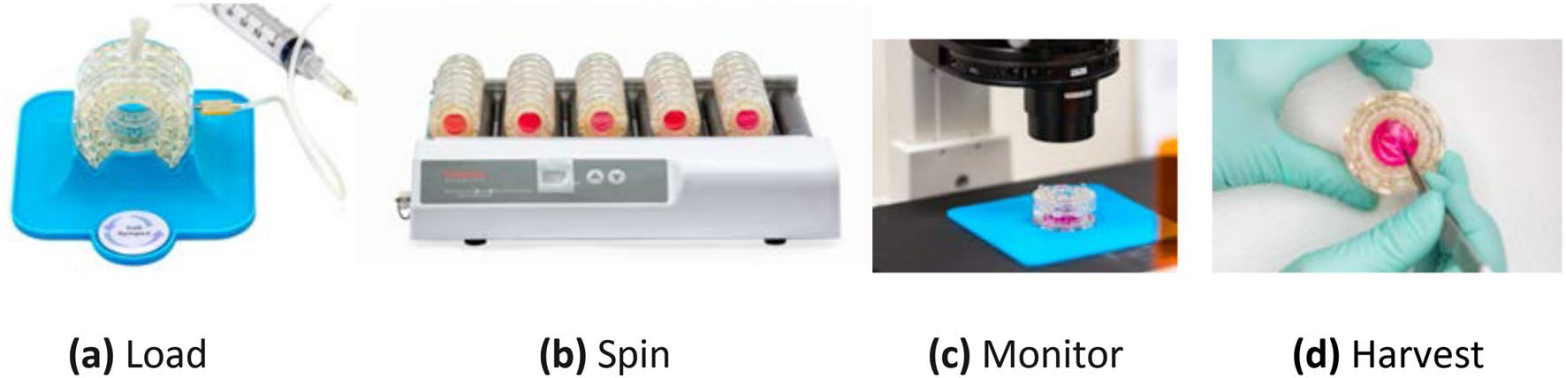
Loading, spinning, monitoring, and harvesting cell spinpods. (**a**) Cells and media are loaded into the cell spinpod, held in a 3D-printed loading dock (blue), with a needle in one port and an air bleed needle in the second self-sealing port. (See also Supplemental video 2). (**b**) Cell Spinpods are spun on an ordinary laboratory bottle roller so that contents are maintained under laminar flow conditions. (See also Supplemental video 1). (**c**) Cells can be monitored in situ still within the Cell Spinpod by microscopy through the optically clear gas exchange membranes when placed in the microscopy holder (blue), which places the cell spinpod’s cellular contents in the focal length of most inverted microscopes. (**d**) Cells can be harvested by incising the membrane or through a needle inserted in the ports. Photographs and videos were taken by author HB.

Cell spinpods are injection-molded polystyrene and polycarbonate cylinders with a 3.5 mL capacity (Fig. 1). The cell chamber has an internal diameter of 19 mm and the overall cell spinpod has an outside diameter of 44 mm. Two self-sealing silicone ports embedded in the rotating rim allow sample loading/removal from one port with simultaneous air bleeding from the other port (Fig. 1a). The cell chamber is sandwiched between two translucent fluoropolymer membranes that retain water but freely exchange oxygen and carbon dioxide (Fig. 1a). The gas /vapor permeability (in g/100 in^2^·24 h.) is 0.4 for water but 1671 for carbon dioxide, and 2200 for oxygen. The cell spinpods are rotated on a laboratory bottle roller, such as those marketed by Thermo-Fisher (Waltham MA), in a 5% CO_2_ incubator (Fig. 2b).

### Two fluid modeling of laminar flow and fluid shear stresses inside the cell spinpod

The fluid mechanical forces experienced by cells in the cell spinpod were simulated based on the two-fluid-model recently proposed by Municchi et al^33^. Verified simulations were performed matching the specifications of the cell cultures and cell spinpods as described herein. Municchi et al. have recently proposed a new computational simulation approach based on the two-fluid model (TFM), in which both the particle and fluid phases of a suspension are considered as interpenetrating continua with their own conservation of mass and momentum equations^33^. The TFM is able to accurately predict particle migration and the non-uniform distribution of suspension stresses. Unlike simpler diffusive flux models used in the literature to model flow and stresses in rotating wall vessels (RWVs)^34^, the TFM does not require the assumptions of a steady suspension velocity and a Stokesian (inertia-less) fluid. Furthermore, the TFM formulation of Municchi et al.^33^ employs suitable anisotropic rheological models to enable simulation of suspension dynamics in general unsteady curvilinear flows, such as those encountered inside cell spinpods. The TFM is implemented using the OpenFOAM^®^ open-source C+ +  platform (www.openfoam.org)^35^.

### Cells

We used Renal Proximal Tubular Endothelial cells immortalized with human Telomerase Reverse Transcriptase, RPTEC/TERT1 cells (ATCC CRL-4031, Manassas, VA, USA) as they show characteristic morphological and functional properties of PTC; e.g. formation of tight junctions and domes, expression of aminopeptidase N, cAMP induction by parathyroid hormone, sodium-dependent phosphate uptake, and an active megalin/cubilin transport system^36^. Equally important, no genomic instability has been observed for up to 90 population doublings^36^. Frozen aliquots of cells were thawed and expanded as monolayer cultures in Dulbecco's Modified Eagle Medium/F12 (DMEM/F12), supplemented with ‘SingleQuots’ containing cell-type specific growth supplements (human epithelial growth factor, insulin, hydrocortisone, transferrin, fetal calf serum, triiodothyronine, epinephrine, and gentamicin-amphotericin; Lonza, Morrisville, NC, USA)^10,37^. Cells were cultured in a 37 °C incubator with a 5% CO_2_ humidified atmosphere and passaged when 70% confluent. Before loading into cell spinpods, trypsinized renal cells at 0.5 × 10^6^ cells/mL were allowed to attach to a suspension of preswollen Cytodex-3 collagen microcarrier beads (25 mg/mL, average diameter 175 µm; Sigma-Aldrich, St. Louis, MO, USA) for one hour at 37 °C. The cell/bead mixture was adjusted to a final concentration of 5 × 10^4^ cells/mL with 2.5 mg/mL beads for loading into the cell spinpod^10,37^.

### Cell spinpod loading and harvest

3.5 mL of renal cells on Cytodex-3 beads, representing approximately 1.75 × 10^5^ total cells, were inoculated into each cell spinpod by gravity flow using a 5-mL syringe and 18-gauge needle. A 27-gauge needle inserted in the second port allowed the escape of air during loading (Fig. 2a; Supplemental Video S2).

The cell spinpods were rotated at 30 revolutions per minute on a bottle roller in a 5% CO_2_ incubator (Fig. 2b). The Bonn criteria are a standardized template for defining the conditions of rotating suspension cultures^38^. For cell spinpods in our experiments, the Bonn criteria are as follows: cell chamber diameter of 1.9 cm, rotation speed of 30 rpm, media viscosity of 0.78 centipoise, media density of 1 g/mL, cell diameter of 12 microns, cell density of 1.05–1.07 g/cm^3^; 175 micron spherical Cytodex-3 beads with a density of 1.04 g/cm^3^ and zero porosity.

Cell spinpods were rotated continuously for 0 to 72 h, as noted in individual experiments. As controls, static cell spinpods were laid flat and maintained stationary in the CO_2_ incubator for the same period of time. At the end of the experiment, the cells and media were harvested by incising the membrane with a scalpel blade. For gene expression pathway analyses, the cell pellet was snap frozen in liquid nitrogen prior to library preparation for next generation sequencing and analysis by Gene Set Enrichment Analysis (GSEA) and Cytoscape visualization. For flow cytometry studies, the cell/bead pellet was washed once with 3 mL of phosphate buffered saline and incubated with 200 µL trypsin–EDTA (0.05% trypsin and 0.02% EDTA) for 120 s at 37 °C to detach the cells from the beads. Trypsinization was stopped with the addition of 100 µL fetal bovine serum, and the cells were again washed with 3 mL of phosphate buffered saline.

### Microscopy of cells in cell spinpod loading and harvest

To visualize RPTEC/TERT1 in the cell spinpods, 3 µL of 1 mg/mL propidium iodide (PI) and 3 µL of CyQUANT reagent (ThermoFisher) were injected through the silicone port and mixed by gentle rocking of the cell spinpod. Cells were visualized by inverted microscopy in situ using a specialized holder to bring the cell spinpod contents into the proper focal plane (Fig. 2c).

### Flow cytometric analysis of viability and apoptosis

For viability analyses, the cell pellet was resuspended in Annexin-binding buffer (Life Technologies, Carlsbad, CA, USA) and filtered through 70 micron Flowmi^®^ Cell Strainers (Millipore Sigma, St. Louis, MO, USA) to remove beads. 100 µL of cell suspension was stained with 5 µL Annexin V, Alexa Fluor 488 conjugate per manufacturer's instructions (Life Technologies, Carlsbad, CA, USA) and 1 µL of 1 mg/mL PI. Cells were analyzed on a Beckton-Dickenson Accuri C+ flow cytometer using log amplified photomultipliers. With every run, quality control was performed on the instrument with 3 color fluorescent beads, followed by assay of four control tubes: no dyes, annexin V alone (488 nm excitation 533/30 nm emission), PI alone (488 nm excitation 585/40 nm emission), and annexin V plus PI together to set compensation. 2000 events were recorded from each sample and all flow cytometry values are in arbitrary fluorescence units.

### Cytokines and renal damage markers

Supernatants of the cells cultured in cell spinpods were assayed for Neutrophil Gelatinase-Associated Lipocalin (NGAL, also known as Lipocalin-2) as an indicator of renal cell damage, and for the cytokines IL-6 and GM-CSF, using Luminex xMAP technology for multiplexed analyses by Eve Technologies Corp. (Calgary, Alberta, Canada). The baseline quantity of NGAL released by renal cells varied between experiments so, in selected statistical analyses, NGAL results were normalized, by experiment, to the average quantity of NGAL released by media controls in static cell spinpods.

### Endocytic receptor assays and membrane transporter assays

Uptake of albumin and dextran was measured with FITC-conjugated reagents. Clean separation of populations was achieved with 1 mg/mL (final) FITC-dextran for 24 h and 400 µg/mL (final) FITC-albumin for two hours in serum-depleted media. RPTEC/TERT1 cells on Cytodex carrier beads were cultured in cell spinpods under rotating or static conditions. FITC-dextran (1 mg/mL final) was added to cell spinpods with renal cells that had been rotated or held static for 2 days; the rotation or static conditions were continued for an additional 24 h before harvest. For albumin uptake, renal cells were cultured in rotating or static cell spinpods for three days, after which the media was replaced with serum-depleted media (containing 20 µg/mL of albumin) containing FITC-albumin (400 µg/mL final). The cell spinpods were incubated for two more hours under their same rotating or static conditions before harvest^12,39^. Cells from all samples were then trypsinized off the carrier beads, washed, filtered to remove beads, and analyzed by flow cytometry.

Uptake of glucose was assayed with the fluorescent substrate 2-(*N*-(7-nitrobenz-2-oxa-1,3-diazol-4-yl)amino)-2-deoxyglucose (2-NBDG)^40^. Cells from rotating and static cell spinpods were trypsinized off of carrier beads, washed in phosphate buffered saline, as described above, and incubated with 2-NBDG (100 µM, or 500 µM final, as indicated) in 140 mM NaCl, 5 mM KCl, 2.5 mM CaCl_2_, 1 mM MgSO_4_, 1 mM KH_2_PO_4_, and 10 mM HEPES, pH 7.4, at 37 °C for 20 or 60 min as indicated^40^. Uptake of 2-NBDG was quantified as the mean FITC signal by flow cytometry. To determine the active sodium-dependent component of the glucose uptake, assays were also conducted in sodium-free buffer in which sodium chloride was replaced by choline chloride and values are presented as net uptake above the non-specific binding control, measured by adding cold NBDG to renal cells on ice immediately before analysis.

### Mitochondrial membrane potential

Mitochondrial membrane potential was measured with the cationic, lipophilic indicator dye JC-10, which is concentrated in the mitochondria and forms reversible red-fluorescent JC-10 aggregates in cells with polarized mitochondrial membranes, but reverts to its monomeric green fluorescent form with the loss of mitochondrial membrane potential (per manufacturers' instructions—Mitochondrial Membrane Potential kit, Sigma Aldrich, St. Louis, MO, USA). Cells from rotating and static cell spinpods were trypsinized off of carrier beads, washed in phosphate buffered saline, as described above, and incubated with JC-10 dye for 15 min at 37 °C and read without washing. JC-10 was diluted in kit buffer A, but optimized at ten-fold more dilute than called for in the manufacturer's directions. Data is reported as the ratio of mean fluorescence intensity in the PerCP channel (red) divided by mean fluorescence intensity in the FITC channel (green). To validate the membrane potential changes, the effects of inhibition of electron transport chain complex I, with 0.5 µM rotenone, and complex III, with 0.5 µM antimycin A, inhibition of ATP production with 2 µM oligomycin, or exposure to the oxidative phosphorylase un-coupler 2 µM 2-[2-[4-(trifluoromethoxy)phenyl]hydrazinylidene]-propanedinitrile (FCCP) were measured by spectrophotometry of JC10 loaded renal cells^41^. For spectrophotometry assays, MMP was calculated as the ratio of red fluorescence (540 nm excitation/590 nm emission) to green fluorescence (492 nm excitation/535 nm emission) and data is presented as the percent of MMP signal after addition of the test reagent relative to the initial baseline.

### Xenobiotic efflux transporter assays

The function of the xenobiotic transporters ABCG2 (also known as BCRP), ABCB1 (also known as Pgp and MDR1) and/or the ABCC2/4 (also known as MRP2/4), was evaluated with 5-chloromethylfluorescein diacetate (CMFDA) in the presence and absence of the inhibitor MK571 ((L-660711, 5-(3-(2-(7-Chloroquinolin-2-yl)ethenyl)phenyl)-8-dimethylcarbamyl-4,6-dithiaoctanoic acid sodium salt hydrate), CMFDA diffuses into cells where it undergoes hydrolysis and glutathione conjugation to carboxyfluorescein-glutathione (GS-MF). GS-MF is transported out of cells by ABCG2, ABCB1, and ABCC2/4, all of which are inhibited by MK571^42^. Renal cells were cultured in cell spinpods under rotating or static conditions for 48 h, trypsinized as described above, and resuspended in DMEM/F12. Protein-free DMEM/F12 was used throughout the assay. A 1 mM solution of CMFDA in DMSO was diluted to a 75 nM working solution in DMEM/F12 and used at a final concentration of 12.5 nM. A 50 mM solution of MK571 in DMSO was diluted to a 300 µM working solution in DMEM/F12 and used at a final concentration of 50 µM. An unstained aliquot of cells from each cell spinpod was placed on ice for a determination of nonspecific binding of CMFDA by adding chilled CMFDA to the iced cells and immediately analyzing the sample by flow cytometry. The remaining cells were incubated with CMFDA for 40 min at 37 °C, washed once with 3 mL of DMEM/F12, and resuspended in fresh DMEM/F12. An aliquot of each sample was placed on ice immediately after washing for determination of CMFDA uptake. The remaining sample was divided in half and MK571 added to one set. Efflux of GS-MF was allowed to proceed for 30 min at 37 °C before analysis. Cells were passed through a 70 micron mesh to remove aggregates before analysis by flow cytometry to quantify the cell-associated CMFDA/GS-MF.

### Myeloma light chains

Myeloma free light chains were purified from the urine of patients who had multiple myeloma, light chain proteinuria, and clinical evidence of significant renal damage, using ammonium sulfate precipitation and Sephadex chromatography as described previously^43^. The purity and identity of the myeloma light chains were confirmed by SDS-PAGE and Western blotting and all specimens were determined to be endotoxin-free by Liumulus ameboecyte assay. Myeloma light chains were stored in lyophilized form until dissolved in tissue culture media and sterile-filtered before addition to cells. Myeloma light chains from six donors were evaluated, in serial dilutions, for their toxic effects on renal cells, in static adherent cultures using 96-well plate format. Cell proliferation was measured with CyQUANT (ThermoFisher) using manufacturer's protocol. The isolation and use of the myeloma light chains was approved by the IRB at the Tulane Office of Human Research Protection (IRB reference no. 848169). The experiments were performed in accordance with relevant guidelines and regulations, informed consent was obtained from all participants and/or their legal guardians, and all protected health information was deidentified.

### Doxorubicin and cisplatin nephrotoxins

RPTEC/TERT1 cells were cultured in cell spinpods under rotating and static conditions for two days in the presence of 5 µM doxorubicin, 100 µM cisplatin, or no drug controls. Glucose and FITC-albumin uptake assays were conducted as described above.

### Gene set enrichment (GSEA) analysis

Triplicate samples of rotating and static cell spinpods with RPTEC/TERT1 cells on Cytodex carrier beads were harvested after 0, 3, 24, and 72 h and each cell pellet, containing cell-bound beads, was snap frozen for RNA-Seq. Total RNA was extracted using the Qiagen (Hilden, Germany) RNA easy protocol, followed by genomic DNA removal using Ambion turbo DNase columns (Thermo-Fisher). RNA-seq libraries were prepared using the MGIEasy RNA library prep set (Complete Genomics Inc. San Jose, CA, USA) procedure and a minimum of 20 million mapped reads were collected for each sample on an MGI-200 sequencer using the 2X100 paired-end protocol. Reads were mapped to the human genome (UCSC hg38) using TopHat2^44^ and the transcripts were quantified using Cufflinks^45^. Differential expression (DE) analysis was performed using CuffDiff2^46^. Only genes with “OK” status were retained and ranked by Log2(fold change state 1/state2) (Supplemental Table S1). GSEA v.4.0.0 was used to perform the Gene Set Enrichment Analysis^47^. Enrichment maps were visualized in Cytoscape v.3.8.2 using the EnrichmentMap application v.3.3.2 (https://Cytoscape.org)^48–50^. Nodes with an FDR (false discovery rate) *q*-value = 0, and a combined co-efficient > 0.5 were represented in the final map (Supplemental Table S2). Overlapping nodes with the top NES (Normalized Enrichment Score) values (> 3.5 or < − 3.5) were clustered and labelled together using the AutoAnnotate application v.1.3.4 in Cytoscape (https://Cytoscape.org)^51^. for Supplemental Figs. S1 and S2.

### Statistical analyses

Statistical analyses for the Next Generation Sequencing data is discussed in the methods section relevant to that methodology. For the remaining data, error bars in figures are the mean ± standard error of the mean (SEM) for the indicated number of replicates. Differences in the distributions between groups were considered statistically significant when *p* < 0.05. *P *values were calculated with a Mann–Whitney *U* nonparametric one-tailed test, using continuity correction and a 95% confidence interval for µ (https://astatsa.com/WilcoxonTest/). Statistics are reported as the value of *W* from the Mann–Whitney *U* test, the sample sizes for *n*_*1*_ and *n*_*2*_, and the calculated one-tailed *p* value.

## Results

### Cell spinpod design

The cell spinpod design was created using 3D modeling software. Stereolithography (SLA) 3D printing was used for development of prototypes for multiple CAD configurations. Each configuration was tested and modified until an optimal sealing condition was obtained, based on empirical testing. Once finalized, the parts were produced by injection molding, assembled, and radiation-sterilized as single-use, disposable, vessels for rotating suspension culture.

The cell spinpod is a polystyrene and polycarbonate cylinder with a 3.5 mL capacity (Fig. 1). Two self-sealing silicone ports are embedded in the rotating rim to allow sample loading/removal from one port with simultaneous air bleeding from the other port (Fig. 1a) (Video S2 in Supplemental data). The cell chamber is sandwiched between two translucent Fluoropolymer membranes that retain water but freely exchange oxygen and carbon dioxide. Gas-exchange was documented as a pH change, i.e. through the change from a characteristic salmon-color of phenol red-containing media in 5% CO_2_ atmosphere to the more purple color in room air. The adequacy of gas exchange was further validated by the ability of the cell spinpods to support the growth of cells, as shown below. In our tests, over three hundred cell spinpods have been filled with media and cultured for three or more days without any evidence of contamination or leakage.

The cell spinpods are rotated on a laboratory bottle roller in a 5% CO_2_ incubator (Fig. 2b). A standard laboratory bottle roller can hold at least 40 cell spinpods. As it rotates, the cell spinpod provides continuous sedimentation of particles through the medium while rotating as a solid mass in laminar flow with regulatable, physiologic levels of induced cellular shear and with no turbulence, as detailed below^5,20,29,52,53^ (see Video S1 in Supplementary Data).

Because the breathable membranes on both sides of the cell spinpod are optically clear, the contents of the cell spinpod can be monitored in situ by microscopy, without removal of the sample from the culture vessel (Fig. 2c, 4). This allows continual monitoring over the course of several days. At the conclusion of the experiment, the cells can be harvested by incising the membrane with a scalpel blade (Fig. 2d), or by aspiration with a needle through the silicone port.

### Laminar flow and fluid shear stresses inside the cell spinpod

**Figure 3:**
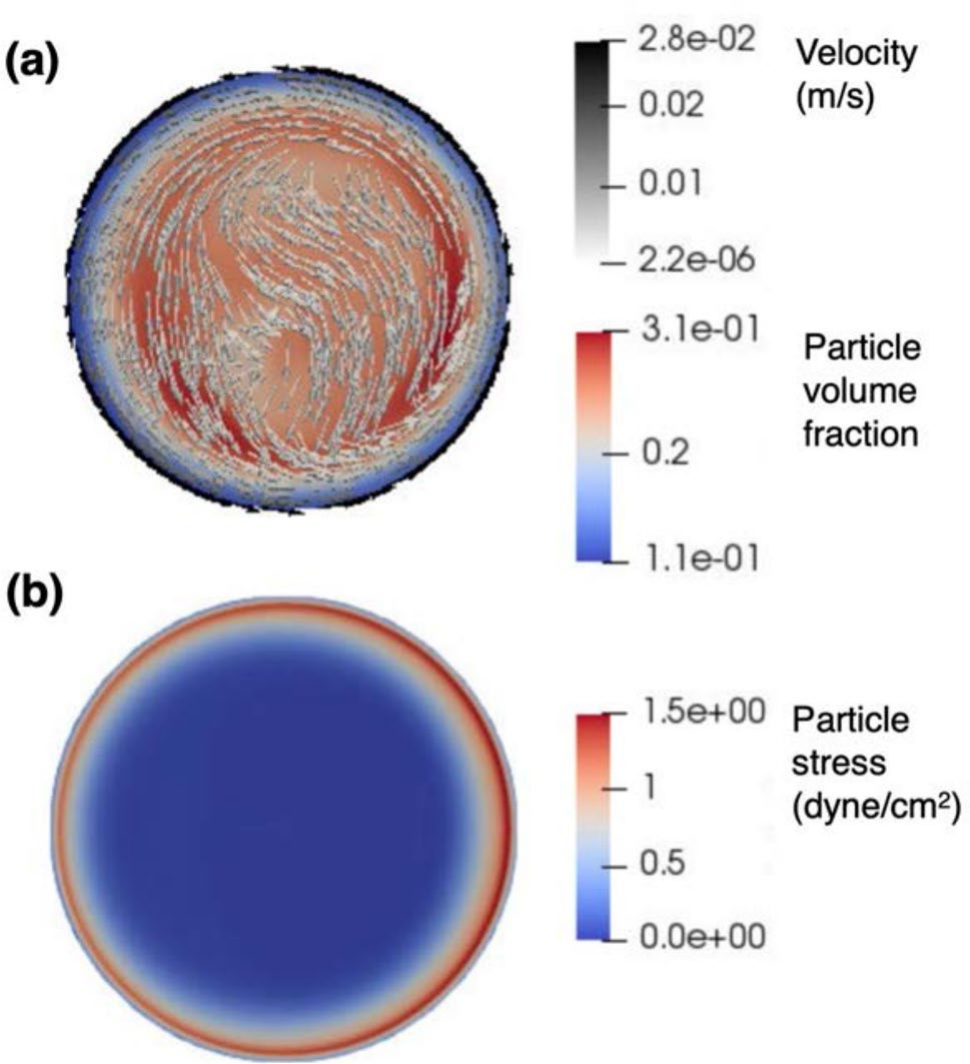
Simulations of the flow and stresses in the cell spinpod. Two fluid model simulations of particle distribution and shear stress in cell spinpod. The flow of a suspension of particles of ${\mathrm{d}}_{\mathrm{p}}=175\mathrm{ \mu m}$ diameter, at an initially homogeneous volume fraction ${\upphi }_{0}=0.2$, was placed in the cylindrical space between two co-centric disks. The suspending fluid was taken to be water at standard conditions. The outer cylinder was rotated at $\upomega =17$ rpm until the suspension reached a steady state (about 500 s later). The suspension’s spatial volume fraction distribution, in an axial cross-section (i.e., perpendicular to the $\mathrm{z}$-axis of rotation out of the page) at steady state is shown by the filled contours in the background of panel (**a**). Panel (**a**) further shows the superimposed velocity vectors color coded by magnitude. Panel (**b**) shows the corresponding distribution of the magnitude of the particle stress tensor in the same plane.

Figure 3 shows the calculated distribution of particles and the fluid shear stress they encounter in a cell spinpod once steady state laminar flow has been achieved (about 500 s after the onset of rotation). Cells are not uniformly distributed throughout the chamber but rather tend to travel in an annular path. Figure 3a shows the volume fraction of particles and velocity vectors illustrating the suspension flow and the distribution of the magnitude of the deviatoric stress tensor of the particle phase. The plots shown are in a cross-section perpendicular to the rotation axis, having first achieved a steady state in the simulation upon starting the rotation from rest. Figure 3b shows the shear stress on the particles. The highest stresses on the particle phase are encountered near the vessel wall (strongest shear) but rapidly decrease to a level of about 0.5 dynes/cm^2^ in the annular region slightly inward from the wall, wherein the volume fraction of particles is highest ($\approx 30\%)$.

### Cell viability and apoptosis in cell spinpods

**Figure 4:**
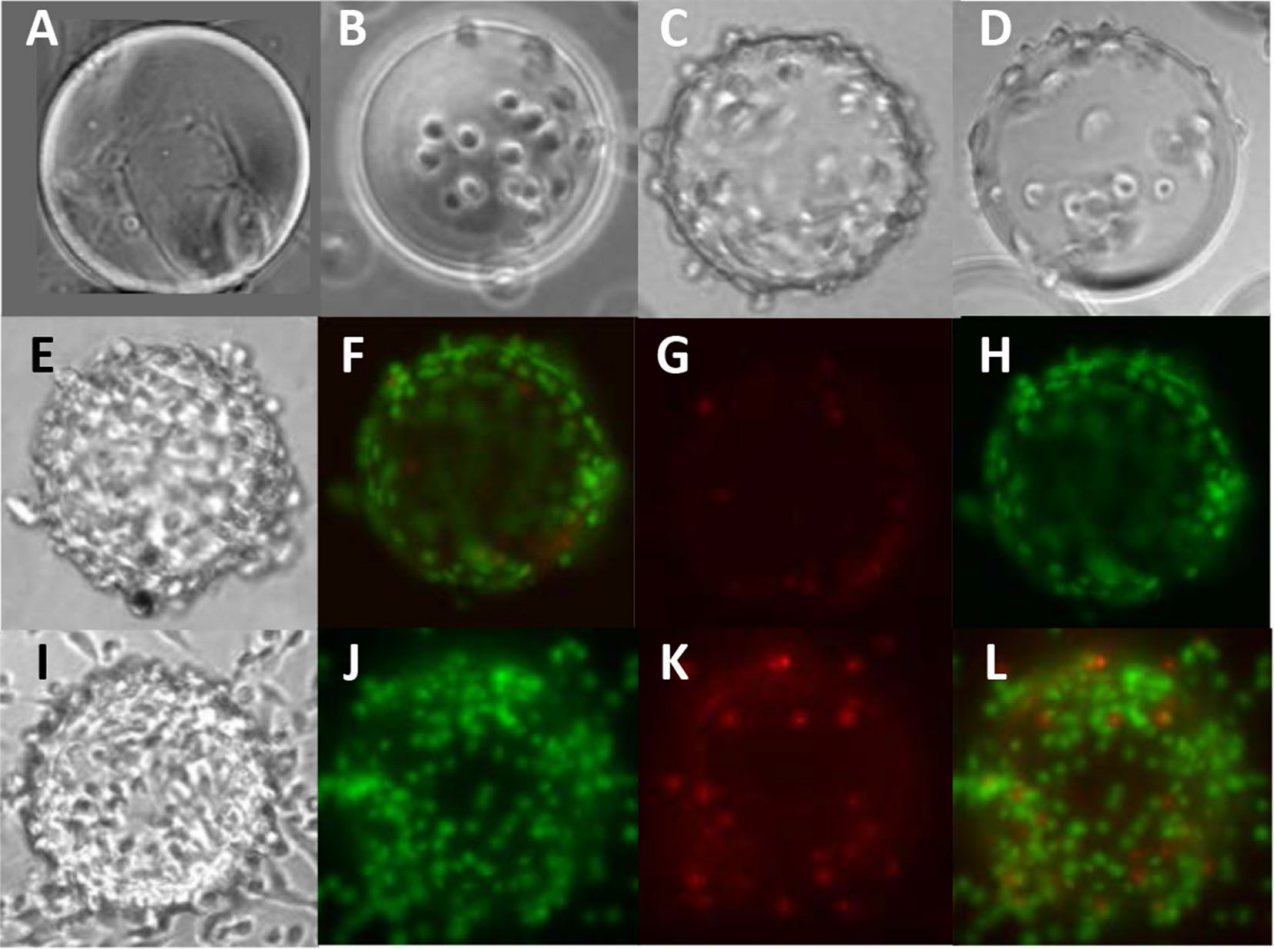
Images of RPTEC/TERT1 in cell spinpods. RPTEC/TERT1 on microcarrier beads were cultured in rotating or static cell spinpods for 48 h after which PI, which is taken up by dead cells, and CyQUANT, a FITC-based reagent that stains all cells, were injected into the cell spinpods to stain the cells in situ. Panel (**a**) shows a 175 micron Cytodex-3 microcarrier bead without cells. Panel (**b**) is a bright field image of a cell spinpods on day 0, immediately after loading renal cells that have incubated 1 h with carrier beads. Panels (**c**) and (**d**) are bright field images of cell spinpods on day 1, rotating and static, respectively. Panels (**e**) through (**h**) are from a rotating cell spinpod and panels (**i**) through (**l**) are from a static cell spinpod on day 2. Panels (**e**) and (**i**) are the bright field images. Panels (**f**) and (**j**) are the FITC images. Panels (**g**) and (**k**) are the PI images. Panels (**h**) and (**l**) are the fused FITC and PI images. Magnification 100×. Photographs were taken by author HB.

The selected inoculum of cells and beads allowed the cells to form a uniform monolayer coating the Cytodex carrier beads by the second day of culture (Fig. 4e–l). Our previous experience had shown that higher loading densities resulted in formation of large aggregates of cells and beads, further enhancing cell polarity and tubule formation. The appearance of renal cells in static and rotating cell spinpods on day 3 is not different, by microscopy, from cell spinpods on day 2.

**Figure 5:**
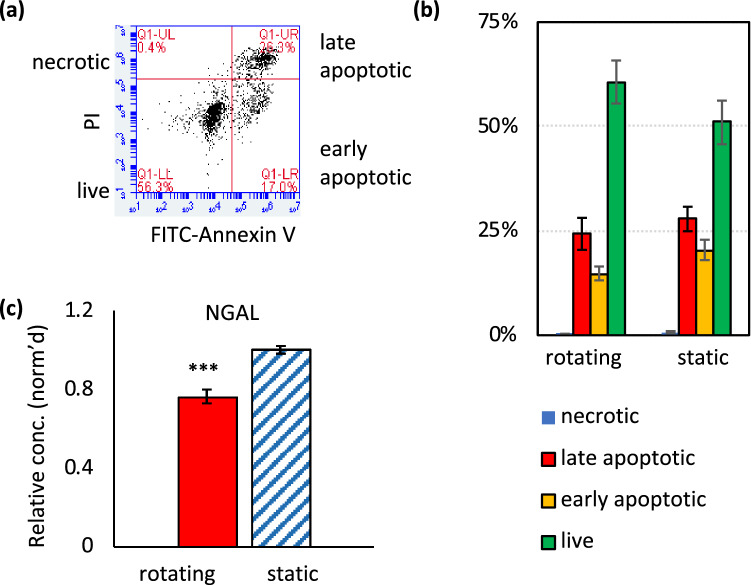
Effect of rotation in cell spinpods on viability of renal cells. RPTEC/TERT1 cells on Cytodex carrier beads were cultured in cell spinpods under rotating or static conditions. After 2 days, cells were trypsinized off the carrier beads, stained with propidium iodide (PI) and FITC annexin V, and analyzed by flow cytometry, panel (**a**). Necrotic cells are in the upper left quadrant (PI^pos^/Annexin^neg^), late apoptotic cells in the upper right quadrant (PI^pos^/Annexin^pos^), early apoptotic cells are in the lower right quadrant (PI^neg^/Annexin^pos^), and live cells are in the lower left quadrant (PI^neg^/Annexin^neg^). Panel (**b**) shows the proportion of cells from rotating and static cell spinpods that are necrotic, in late apoptosis, in early apoptosis, or live. Error bars indicate ± SEM of six replicates. Results are representative of four experiments. Panel (**c**) shows the relative quantity of NGAL released by RPTEC/TERT1 cells in rotating () or static () spin pods. NGAL data have been normalized to the average quantity of NGAL released by cells in static pods for each experiment and error bars indicate ± SEM of data pooled from five experiments. Asterisks indicate where differences in the distributions between groups were statistically significant, e.g. *p* < 0.05, by one-tailed Mann–Whitney *U* test. Rotation in a cell spinpod increased the quantity of NGAL release (*p* = 1.3×e–7, *w* = 83; *n*_1_ = 30, *n*_2_ = 28) but did not induce a significant change in the quantity of PAI released (*p* = 0.38, *W* = 425; *n*_1_ = 30, *n*_2_ = 27).

Flow cytometry was used to measure binding of annexin V to identify apoptotic cells, and uptake of PI to identify dead cells (Fig. 5a). Exposure to fluid shear stress in rotating cell spinpods did not change cell viability or the fraction of cells in early or late apoptosis compared to static cell spinpods (Fig. 5b). However, renal cells cultured in rotating cell spinpods released 76.4 ± 3.5% as much NGAL, a known kidney injury marker^54,55^ compared to renal cells in static spinpods (*p* = 1.3 × e−7, *W* = 83, *n*_1_ = 30, *n*_2_ = 28, Fig. 5c.

### Endocytic receptor function, membrane transporter function, and mitochondrial membrane potential of renal cells in cell spinpods

**Figure 6:**
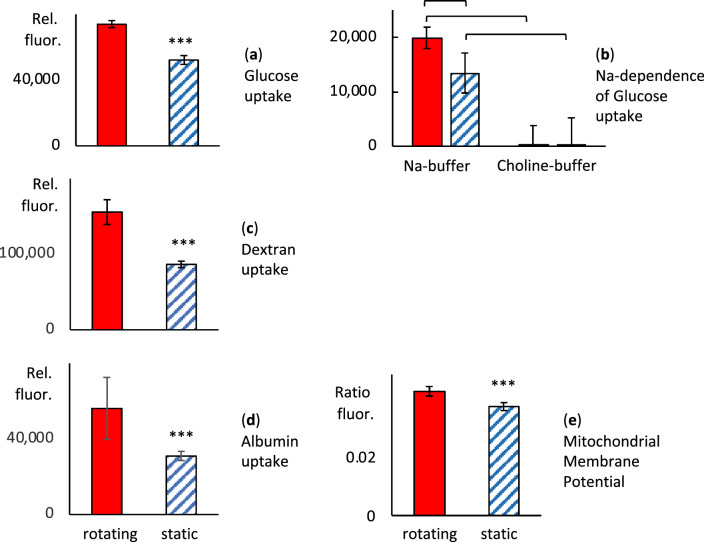
Effect of rotation in cell spinpods on endocytic receptor function, membrane transporter function, and mitochondrial membrane potential. RPTEC/TERT1 cells on Cytodex carrier beads were cultured in cell spinpods under rotating conditions () or static conditions () for 2 days. Panel (**a**) shows the uptake of glucose, measured with the fluorescent substrate 2-NDBG. Panel (**b**) shows the uptake of glucose in Na-containing versus Na-free choline buffer, Panel (**c**) shows the uptake of FITC-dextran. Panel (**c**) shows the uptake of FITC-albumin. Panel (**d**) shows the mitochondrial membrane potential measured with JC10. Cells from all samples were trypsinized off the carrier beads before analysis by flow cytometry. Data is presented as mean fluorescence, in relative fluorescence units, for panels (**a**–**c**) and as the ratio of red:green fluorescence in panel (**d**). Error bars are ± SEM of six replicates. Asterisks or brackets indicate where differences in the distributions between groups were statistically significant, e.g. *p* < 0.05, by one-tailed Mann–Whitney *U* test. Rotation in a cell spinpod induced a statistically significant increase in the uptake of glucose (*p* = 0.001, *W* = 36, *n*_1_ = *n*_2_ = 6,), dextran (*p* = 0.002, *W* = 36; *n*_1_ = *n*_2_ = 6), and albumin (*p* = 0.02, *W* = 31; *n*_1_ = *n*_2_ = 6). Rotation also increased the mitochondrial membrane potential (*p* = 0.018, *W* = 31, *n*_1_ = *n*_2_ = 6).

The increased functionality of renal cells exposed to fluid shear stress in rotating cell spinpods was demonstrated by the increased uptake of glucose, albumin, and dextran as well as increased mitochondrial membrane potential (Fig. 6). Rotation in a cell spinpod significantly increased the uptake of glucose (rotating 73,966 ± 2176 vs. static 52,174 ± 2660 relative fluorescence units (*p* = 0.001, *W* = 36, *n*_1_ = *n*_2_ = 6, Fig. 6a). The glucose uptake was time- and dose-dependent. Extending the incubation time from 20 to 60 min led to a 2.88 ± 0.09 fold-increase in fluorescence signal for cells in rotating conditions (*p* = 0.004, *W* = 0, *n*_1_ = *6, n*_2_ = *5*) and a 2.81 ± 0.18 fold-increase for cells in static conditions (p = 0.002, *W* = 0, *n*_*1*_ = *n*_*2*_ = 6). A five-fold increase in the quantity of NDBG added led to a 2.50 ± 0.13 fold-increase in fluorescence signal for cells in rotating conditions (*p* = 0.001, *W* = *0, n*_1_ = *n*_2_ = 6) and a 2.44 ± 0.18 fold-increase for cells in static conditions (*p* = 0.003, *W* = 0, *n*_1_ = *n*_2_ = 5). Removal of sodium abrogated the glucose uptake confirming that the results reflected active Na-dependent glucose transport (Fig. 6b).

Rotation in a cell spinpod doubled the uptake of 4000 MW FITC-dextran (rotating 155,542 ± 16,361 vs. static 86,347 ± 4335 relative fluorescence units (*p* = 0.002, *W* = 36; *n*_1_ = *n*_2_ = 6, Fig. 6c). Rotation also doubled the uptake of FITC-albumin (rotating 56,153 ± 16,350 vs. static 30,976 ± 2402 relative fluorescence units, *p* = 0.02, *W* = 31; *n*_1_ = *n*_2_ = 6, Fig. 6d). These results suggest that rotation in a cell spinpod increased the activity of megalin, the receptor known to be responsible uptake of albumin and dextran^56,57^.

Exposure to fluid shear stress in a rotating cell spinpod also increased the mitochondrial membrane potential (red:green fluorescence ratio for rotating 0.044 ± 0.002 vs. static 0.039 ± 0.001, (*p* = 0.018, *W* = 31, *n*_1_ = *n*_2_ = 6, Fig. 6e). To validate the mitochondrial membrane potential data, we evaluated the effect of uncouplers that dissipate the potential measured as the ratio of ratio of red fluorescence (Ex 540/Em590) to green fluorescence (Ex 492/Em 535). Oligomycin, which inhibitions ATP production, reduced the MMP signal to 70.8 ± 2.5% of baseline (*p* = 0.001, *W* = 36, *n*_1_ = *n*_2_ = 6). FCCP, a protonophore that collapses the proton gradient, reduced the MMP to 11.7 ± 2.0% of baseline (*p* = 0.001, *W* = 36, *n*_1_ = *n*_2_ = 6). Antimycin A, which inhibits electron transport chain complex III, plus rotenone, which inhibits complex I, reduced the MMP to 56.3 ± 2.9% of baseline (*p* = 0.004, *W* = 2, *n*_1_ = *n*_2_ = 6).

### Xenobiotic transporter activity of renal cells in cell spinpods

**Figure 7:**
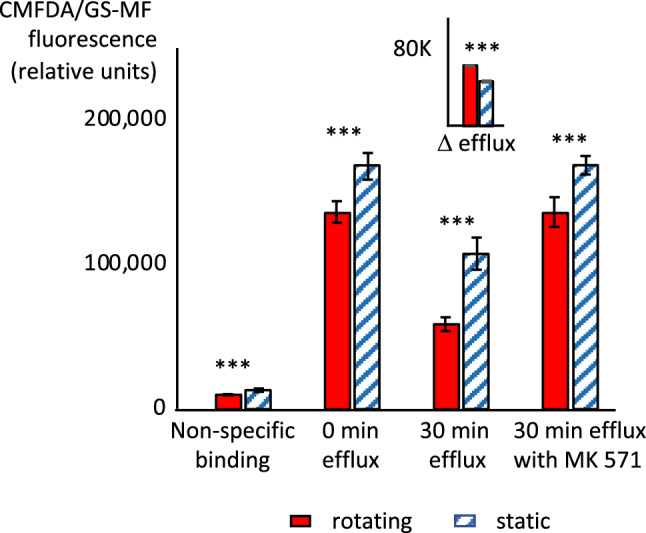
Effect of rotation in cell spinpods on the activity of xenobiotic efflux transporters. RPTEC/TERT1 cells on Cytodex carrier beads were cultured in cell spinpods under rotating () or static conditions () for 48 h and harvested. One aliquot of cells was chilled and cold CMFDA was added before immediate analysis to measure non-specific binding. The remaining cells were incubated with CMFDA for 40 min at 37 °C, washed, and the CMFDA/GM-SF was allowed to efflux in the presence and absence of the inhibitor MK571. The quantity of CMFDA/GS-MF in the cells was measured by flow cytometry immediately after washing (0 min efflux) and after 30 min of efflux at 37 °C (30 min efflux and 30 min efflux with MK 571). Asterisks indicate where differences in the distributions between groups were statistically significant, e.g. *p* < 0.05, by one-tailed Mann–Whitney *U* test. Non-specific binding was significantly higher in cells from rotating cell spinpods (*p* = 0.00015, *W* = 2, *n*_1_ = *n*_2_ = 5). CMFDA/GS-MF remaining in cells from rotating cell spinpods was significantly lower at time zero (*W* = 4, *n*_1_ = *n*_2_ = 6, *p* = 0.013), after 30 min of efflux (*p* = 0.00035, *W* = 0, *n*_1_ = *n*_2_ = 5), and after 30 min of efflux in the presence of MK571 (*p* = 0.065, W = 6, *n*_1_ = *n*_2_ = 6). The inset shows the difference in CMFDA/GM-SF signal between time 0 and after 30 min of efflux. The loss of CMFDA/GM-SF from cells in rotating cell spinpods was significantly larger (*p* = 0.008, *W* = 24, *n*_1_ = *n*_2_ = 5).

The increased functionality of renal cells exposed to fluid shear stress in rotating cell spinpods was also demonstrated by the increased activity of xenobiotic transporters (Fig. 7). CMFDA diffuses into cells where it is conjugated to carboxyfluorescein-glutathione (GS-MF). CMFDA and GS-MF are actively transported back out of the cells by three transporters: ABCG2 (also known as BCRP), ABCB1 (also known as Pgp), and ABCC2/4^42^. Cells from rotating cell spinpods contained significantly lower FITC-signal at the end of the 40-min loading interval compared to cells from static cell spinpods reflecting the increased activity of these three transporters in effluxing the CMFDA/GM-CF out of the cells during the loading phase (rotating 136,465 ± 7509 vs. static 168,067 ± 9241 relative fluorescence units, *p* = 0.01, *W* = 4, *n*_1_ = *n*_2_ = 6, Fig. 7). After washing the free CMFDA away and allowing the transporters to continue to efflux GS-MF out of the cells for 30 more minute, the quantity of CMFDA/GS-MF remaining in cells from rotating cell spinpods was significantly lower than in cells from static cell spinpods (rotating 58,849 ± 4769 vs. static 114,684 ± 10,602 relative fluorescence units, *p* = 0.00035, *W* = 0, *n*_1_ = *n*_2_ = 5). The inset in Fig. 7 shows the additional efflux of CMFDA/GS-MF after the excess dye is was washed away and efflux continues for 30 min more, expressed as the delta in fluorescence. The efflux of CMFDA/GS-MF from cells in rotating spell spinpods was significantly larger (rotating 74,592 ± 6620 vs. static 54,230 ± 1971 relative fluorescence units *p* = 0.008, *W* = 24, *n*_1_ = *n*_2_ = 5,). The efflux of CMFDA/GS-MF was completely abrogated by addition MK571 which inhibits ABCG2, ABCB1, and ABCC2/4^42^ (136,465 ± 7509 relative fluorescence units without inhibitor vs. 136,434 ± 10,254 with MK571 in rotating cultures and 168,067 ± 9241 without inhibitor vs. 168,853 ± 6400 with MK571 in static cultures). These results indicate that exposure to fluid shear stress in the rotating cell spinpods increased the activity of the xenobiotic transporters ABCG2, ABCB1 and/or ABCC2/4 in renal cells.

### Effect of doxorubicin and cisplatin nephrotoxins on membrane transporter function

**Figure 8:**
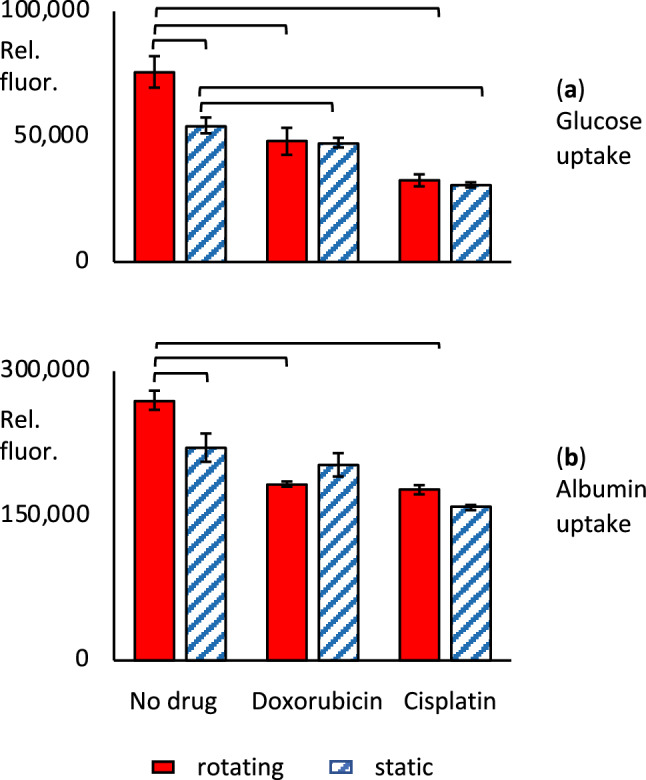
Effect of rotation in cell spinpods on effect of chemotherapeutic agents. RPTEC/hTERT cells on carrier beads were cultured in cell spinpods under rotating conditions () or static conditions () for two days in the presence of 5 µM doxorubicin, 100 µM cisplatin, or no drug control. Panel (**a**) shows the uptake of glucose, measured with the fluorescent substrate 2-NDBG. Panel (**b**) shows the uptake of FITC-albumin. Cells from all samples were trypsinized off the carrier beads before analysis by flow cytometry. Data is presented as mean fluorescence ± SEM of six replicates (except for 5 replicates with static cisplatin), in relative fluorescence units. Brackets mark where differences in the distributions between groups were statistically significant, e.g. *p* < 0.05, by one-tailed Mann–Whitney *U* test.

To evaluate the responsiveness of renal cells to known nephrotoxins, we exposed RPTEC/hTERT cells in cell spinpods to the chemotherapeutic agents doxorubicin and cisplatin and measured the uptake of glucose and albumin after 48 h (Fig. 8). In the absence of drug, rotation in cell spinpods increased the uptake of albumin (269,286 ± 9960 rotating vs. 220,706 ± 14,684 static*,* *p* = 0.05, *W* = 28*, n*_1_ = *n*_2_ = 6) and uptake of glucose (75,848 ± 6269 rotating vs. 54,496 ± 3129 static, *p* = 0.013, *W* = 32, *n*_1_ = *n*_2_ = 6), as we had previously seen. The rotation-induced increased uptake of glucose and albumin was abrogated by 5 µM doxorubicin and by 100 µM cisplatin (Fig. 8). In addition, both drugs caused significant decreases in the quantity of glucose and albumin taken up by renal cells in rotating conditions (Glucose uptake 75,848 ± 6269 with no drug vs. 48,138 ± 5390 with doxorubicin, *p* = 0.015, *W* = 33, *n*_1_ = *n*_2_ = 6, and 32,601 ± 2391 with cisplatin, *p* = 0.002, *W* = 36, *n*_1_ = 6, *n*_2_ = 5. Albumin uptake 269,286 ± 9960 with no drug vs. 182,961 ± 2630 with doxorubicin, *p* = 0.005, *W* = 36, *n*_1_ = *n*_2_ = 6, and 177,099 ± 4722 with cisplatin, *p* = 0.002, *W* = 36, *n*_1_ = 6, *n*_2_ = 5). Cisplatin also reduced the amount of glucose and albumin taken up in static cultures (Glucose uptake 220,706 ± 14,684 with no drug vs 158,589 ± 2720 with cisplatin, *p* = 0.004* W* = 30, *n*_1_ = *6, n*_2_ = 5. Albumin uptake 54,496 ± 3129 with no drug vs. 30,741 ± 1024, *p* = 0.004, *W* = 30, *n*_1_ = 6, *n*_2_ = 5).

### Cytokine release induced by myeloma free light chains

**Figure 9:**
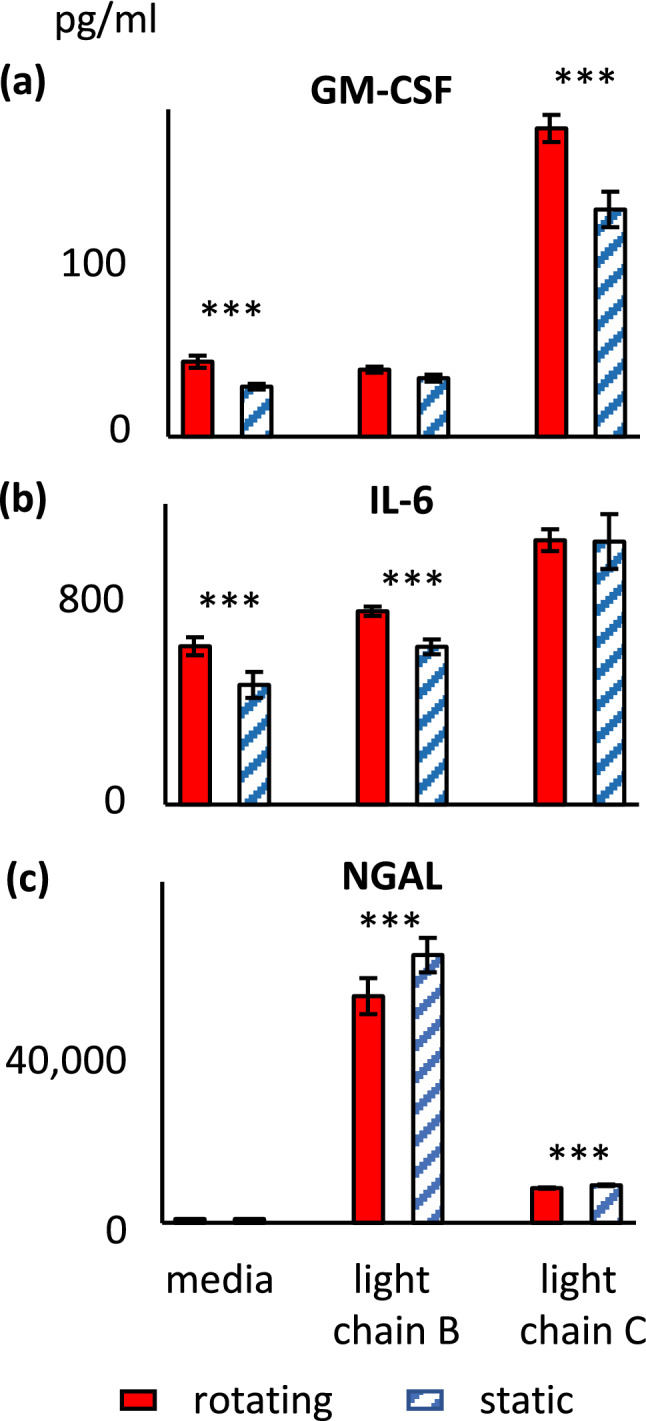
Effect of rotation in cell spinpods on cytokine release from renal cells. RPTEC/TERT1 cells on Cytodex carrier beads were cultured in cell spinpods under rotating conditions () or static conditions () in the presence of myeloma light chains from donor B, myeloma light chains from donor C, or media. After 48 h, supernatants were harvested for assay of cytokines. Bars presented mean concentration (pg/mL) of GM-CSF, panel (**a**), IL-6, panel (**b**), and NGAL panel (**c**), in the cell supernatant; error bars are ± SEM. Asterisks indicate where differences in the distributions between rotating and static groups were statistically significant, e.g. *p* < 0.05, by one-tailed Mann–Whitney *U* test. Rotation induced significantly greater quantities of GM-CSF in the media control (*p* = 0.004, *W* = 39, *n*_1_ = 7, *n*_2_ = 6) and after stimulation with myeloma light chains from donor C (*p* = 0.005, *W* = 33, *n*_1_ = 7, *n*_2_ = 5). Rotation induced significantly greater quantities of IL-6 after culture in media alone (*p* = 0.036, *W* = 34, *n*_1_ = 7, *n*_2_ = 6) and with myeloma light chains from donor B (*p* = 0.001, *W* = 41, *n*_1_ = 7, *n*_2_ = 6). Rotation significantly reduced the quantities of NGAL released after stimulation with myeloma light chains from donor C (*p* = 0.024, *W* = 5, *n*_1_ = 7, *n*_2_ = 5) and myeloma light chains from donor B (Fig. 8c, *p* = 0.05, *W* = 9, *n*_1_ = 7, *n*_2_ = 5).

To further evaluate the response of renal cells in cell spinpods, we exposed them to myeloma light chains, which are a known nephrotoxin^58,59^ and are taken up by megalin^60^. Preliminary evaluation of myeloma light chain toxicity using renal cells in 96-well 2D cultures showed that at a dose of 100 µM, myeloma light chains from donor B caused a 60.0 ± 1.9% reduction in the proliferation of renal cells (370 ± 18 vs. vs. 912 ± 26 relative fluorescence units, *p* = 0.000004, *W* = 0, *n*_1_ = 6, *n*_2_ = 24,) whereas myeloma light chains from donor C did not have a significant toxic effect at that dose (5 ± 5.6% reduction in proliferation; 871 ± 46 vs. 912 ± 26 relative fluorescence units, *p* = 0.77, *W* = 63, *n*_1_ = 6, *n*_2_ = 24). These two donors' myeloma light chains were then evaluated for their effect on renal cells in rotating and static spinpods. As might be predicted from the screening viability studies, myeloma light chains from donor B induced ~ 700-fold increase in the quantity of NGAL released by cells in rotating or static spinpods (Fig. 9c), rotating 30,708 ± 2435 vs. media 42 ± 4 ng/mL and static 36,261 ± 2339 vs. media 53 ± 4 ng/mL) whereas myeloma light chains from donor C induced only ~ 100-fold increase in NGAL release (rotating 4722 ± 89 ng/mL and static 5107 ± 113 ng/mL). Myeloma light chains from donor C also induced a significant increase in the quantity of GM-CSF released by renal cells in rotating and static spinpods (rotating 188 ± 8 vs. media 46 ± 4 ng/mL, *p* = 0.00025, *W* = 0, *n*_1_ = *n*_2_ = 7; static 139 ± 11 vs. 31 ± 2 ng/mL, *p* = 0.002, *W* = 0, *n*_1_ = 6, *n*_2_ = 5, Fig. 9a).

Fluid shear stress also affected cytokine and NGAL release. Rotation induced significantly greater quantities of GM-CSF in the media control (45.8 ± 3.7 ng/mL for rotating vs. 30.6 ± 1.7 ng/mL static; *p* = 0.004, *W* = 39, *n*_1_ = 7, *n*_2_ = 6) and after stimulation with myeloma light chains from donor C (Fig. 9a, 188 ± 8 ng/mL rotating vs. 139 ± 11 static; *p* = 0.005, *W* = 33, *n*_1_ = 7, *n*_2_ = 5,) but not myeloma light chains from donor B (41 ± 2 ng/mL rotating vs. 36 ± 2 ng/mL static). Fluid shear stress increased IL-6 release in renal cells cultured with media alone (632 ± 36 ng/mL rotating vs. 478 ± 52 ng/mL static, *p* = 0.036, *W* = 34, *n*_1_ = 7, *n*_2_ = 6,) and with myeloma light chains from donor B (Fig. 9b, 772 ± 19 ng/mL rotating vs. 630 ± 30 ng/mL static, *p* = 0.001, *W* = 41, *n*_1_ = 7, *n*_2_ = 6), but not myeloma light chains from donor C (1055 ± 44 ng/mL rotating vs. 1040 ± 109 ng/mL static). Fluid shear stress also decreased NGAL release in renal cells cultured with myeloma light chains from donor C (Fig. 9c, 4722 ± 89 ng/mL rotating vs. 5106 ± 113 ng/mL static, *p* = 0.024, *W* = 5, *n*_1_ = 7, *n*_2_ = 5), and myeloma light chains from donor B (Fig. 8c, 30,708 ± 2435 ng/mL rotating vs. 36,261 ± 2339 ng/mL static, *p* = 0.05, *W* = 9, *n*_1_ = 7, *n*_2_ = 5).

### Next generation sequencing

Renal cells exposed to fluid shear stress in rotating suspension cultures upregulate distinct gene pathways compared to renal cells in static cultures. RNA-Seq analysis shows a difference in the identity and timing of gene expression responses of RPTEC/TERT1 cells in cell spinpods when they are static or rotated. Supplemental Table S1 lists the differentially expressed genes and their significance based on the false discovery rate for rotating and static cell spinpods at all time points. Enrichment maps can be generated by comparing changes across time under static or rotating conditions or by comparing static with rotating conditions for each time point. Supplemental Fig. S1 is an enrichment map for rotating and static cell spinpods at time 0 versus 3, 24, and 72 h. Supplemental Fig. S2 is and enrichment map for the comparison of static versus rotating conditions at each time point. Supplemental Table S2 lists the pathways, genes and variables associated with each node shown in the enrichment maps shown in Supplemental Figs. S1 and S2.

Viewed across time, at 3 h of growth, the cells in static cell spinpods already display increased expression of genes involved in RNA gene expression and RNA polymerase biosynthesis, as well as genes involved in changes in apoptotic cell death, response to different stimuli, and intracellular protein acetylation. In contrast, at 3 h of exposure to fluid shear stress in rotating cell spinpods, genes involved in the response to DNA-damage, oxidative stress, cell cycle regulation, and immune response signaling pathways are upregulated. Furthermore, the 3-h rotating cells manifest upregulation of a greater number of genes involved in cellular metabolic processes, which is consistent with our flow cytometry and cytokine data. By 24 h the cells in static cell spinpods manifest large changes in the categories of oxygen compound response, cellular metabolic processes, and apoptotic process regulation. At the same 24-h time period, the rotating cells are also showing changes a great number of genes involved in apoptosis and metabolic processes such as such as protein acetylation. By 72 h, the cells in both static and rotating cell spinpods show changes in oxygen level response in addition to the other major processes observed in other time points. In all states, sensory perception of chemical stimuli is downregulated.

In a direct comparison of rotating versus static cell spinpods, the GSEA processes that differed under fluid shear stress included cell adhesion, cytoskeletal and junctional organization, respiratory chain transport and mitochondrial gene expression, GTPase activity, among others (Table 1).

**Table 1:** GSEA processes in RPTEC/TERT1 that differ between rotating and static cell spinpods after 3, 24, and 72 h of culture.

**3 h**
• Cell junction organization
• Cell adhesion
• Mitochondrial gene expression
• Protein localization to endoplasmic reticulum
• GTPase mediated signal transduction
• Regulation of GTPase activity
• Respiratory transport chain
• Neuron projection development
• Heart development
• Blood vessel morphogenesis
• Camera-type eye development
**24 h**
• DNA replications and repair
• Regulation of cell cycle events
• Autophagy
• Mitochondrial gene expression
• RNA splicing
• Ribosomal biogenesis
• RNA catabolic process and protein targeting to membrane
• Chromosome organization
**72 h**
• Cytoskeleton organization
• Cell adhesion
• Cell junction organization
• Mitochondrial translation
• DNA replication
• Chromosome organization
• DNA geometric changes
• Wound healing
• Tissue morphogenesis
• Brain development
• Response to growth factors
• Regulation of neuron projection development
• Blood vessel morphogenesis

Enrichment maps of this data can be found in Supplemental Fig. S2 and the pathways, genes and variables can be found in Supplemental Table S2.

**Figure 10:**
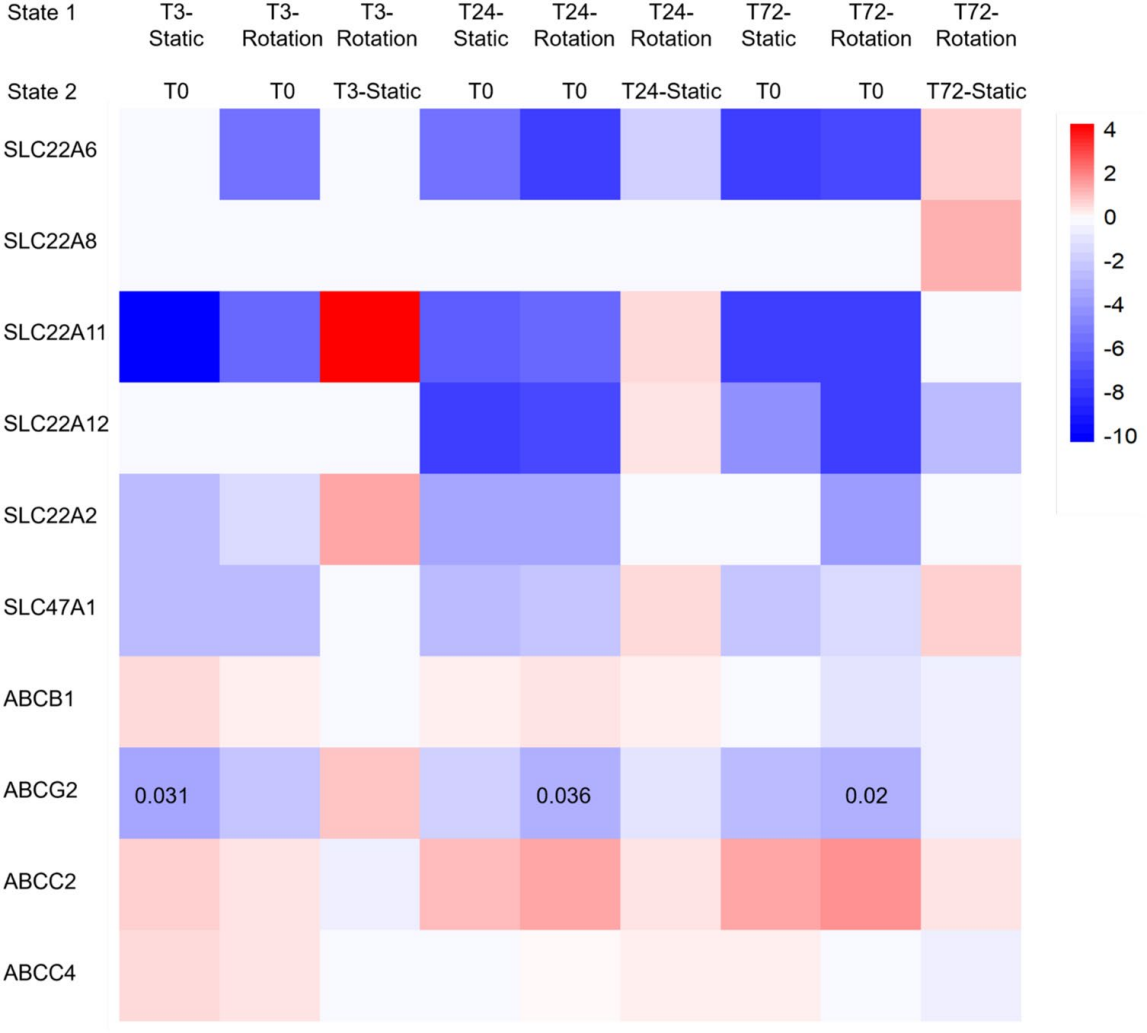
Changes in expression level among 10 renal transporter genes in RPTEC/TERT1 cells, rotation versus static conditions at 3-, 24-, and 72-h time points. Heatmap represents log2(fold change state1/state2) values. The heatmap was created in R v.3.6.2^1^ (https://www.R-project.org/) using package pheatmap v.1.0.12^2^ (https://CRAN.R-project.org/package=pheatmap). FDR *q *values are indicated for the 3 significant differentially expressed states. White color (log_2_(FC) = 0) indicates DE genes with “NOTEST” status, where not enough alignments have been present for testing. Alternate names for the transporters in the heatmap include: organic anion transporter 1 (OAT1: SLC22A6), organic anion transporter 3, (OAT-3: SLC22A8) organic anion transporter 4 (OAT-4: SLC22A11), urate anion exchanger 1 (URAT1: SLC22A12), organic cation transporter 2 (OCT-2: SLC22A2), multidrug and toxin extrusion proteins 1 (MATE1: SLC47A1), multidrug resistance protein 1 (MDR-1: ABCB1, also known as Pgp), breast cancer resistance protein (BCRP: ABCG2), multidrug resistance protein 2 (MRP2: ABCC2), and multidrug resistance protein 4 (MRP4: ABCC4).

We observed changes in absolute expression level at all time points of the well-characterized renal transporters known to be expressed by PTC including Organic Anion Transporter 1 (OAT1: SLC22A6), Organic Anion Transporter 3, (OAT-3: SLC22A8) Organic Anion Transporter 4 (OAT-4: SLC22A11), Urate Anion Exchanger 1 (URAT1: SLC22A12), Organic Cation Transporter 2 (OCT-2:SLC22A2), Multidrug and Toxin Extrusion Proteins 1 (MATE1: SLC47A1), Multidrug Resistance Protein 1 (MDR-1: ABCB1, also known as Pgp), Breast Cancer Resistance Protein (BCRP;ABCG2), Multidrug Resistance Protein 2 (MRP2: ABCC2), and Multidrug Resistance Protein 4 (MRP4: ABCC4) (Fig. 9). The heatmap shows the log_2_(fold change state1/state2) values for comparisons between rotating and static conditions and between time 0 and 3, 24, and 72 h culture. As a class, these transporters were mostly upregulated in the rotating cell spinpods compared to the static state. However, the only one with significant differential expression *q*-values (< 0.05) was ABCG2, which was reduced at 3 h in the static cultures (*q*-value = 0.031), but this reduction was delayed in rotating cultures with differential expression *q*-values of 0.036 at 24 h, and 0.02 at 72 h, respectively (Fig. 10). Also, the multiligand receptors megalin and cubilin, responsible for the constitutive uptake of a vast variety of molecules^56,57^, did not indicate significant differential expression across different time points and the two states.

## Discussion

This study combines two major initiatives. First, it shows that injection molding and new membrane materials allow the production of inexpensive suspension culture devices, known as cell spinpods. Second, we present a use case demonstrating that cell spinpods provide a potent new tool to study renal physiology and pathophysiology, from cell and tissue constructs, through renal tumor culture, both to test interventions, and for studies of nephrotoxicity (Table 2).

**Table 2:** Advantages and uses of the cell spinpod form of rotational cell culture.

• Orders of magnitude less expensive than current commercially available options
• Use small quantities of (often expensive) reagents and cells
• Occupy less space in laboratory incubators
• Are facile to fill and harvest
• Can be used to maintain spheroids in suspension for in situ analysis
• Can be easily re-fed multiple times with minimal disruption in rotation
• Reagents can be added for analysis in situ
• Optically clear membranes allow inspection by microscopy in situ, including fluorescence microscopy
• Provide BSL-2 containment
• Can be engineered to hold as little as 100 µL and > 100 mL
• Can be engineered to include a bubble trap to evacuate bubbles as they form
• Half-filled cell spinpods can serve as miniature roller bottles
• Adaptable to aerobic, anaerobic, and biofilm applications
• Amenable to automated and robotic applications
• Direct application for pathology, chemotherapy, and toxicity studies

Cell spinpods are a new generation of suspension culture devices in which careful design and selection of materials solve many of the problems encountered with previous generation suspension culture devices (Table 2). Cell spinpods are inexpensive, easy to use, maintain controlled levels of shear stress, and allow direct microscopy and fluorometric/ spectrophotometric analyses of cells still within the cell spinpod. A 3.5 mL vessel capacity was chosen as a ‘Goldilocks-size’ to minimize the amount of media and cells (which are often expensive and limiting), yet provide enough sample for phenotyping, proteomics, and genomics. Our first technical innovation is the use of fluoropolymer for the breathable membranes which has better O_2_/CO_2_ exchange, while at the same time, much lower water vapor exchange, than the gas exchange membranes used in the previous generation rotating wall vessels. The low water vapor loss minimizes the formation of bubbles that can disrupt laminar flow^61,62^. Our second innovation takes advantage of the improvement in direct current motors, allowing us to replace the expensive axle rotators and chronometers with inexpensive bottle rollers for a 10- to 25-fold cost reduction. Our third innovation is complete re-design of the filling ports to make them easier to use and automate. The ports are now made of self-sealing silicon rubber and placed axially, obviating the need for cumbersome *en face* 3-way taps or stopcocks (Table 2).

The shear stress applied to cells during zero head space (i.e. vessels completely filled with fluid and no air) suspension culture is determined by gravity, the radius of the particles squared, the difference in density between the cells and the media, and the viscosity of the media^1,6,27,29–32,34^. Changes in these parameters can be utilized to moderate the shear levels delivered during suspension culture. The dependence on gravity has made suspension culture a popular module for space flight studies and their ground-based simulations^6^.

Of note, the speed of the rotation does not affect shear stress—it only affects the diameter of the annular circular path through which the cells move. Increasing the speed increases the diameter of the annulus, until at boundary conditions the cells collide with the outer walls of the rotating vessel^1,34^. However, so long as the annulus remains within the fluid space of the culture chamber, the shear stress remains constant despite small changes in rotation speed. This feature allows for a wide variety of cell sizes and cell spheroids to be cultured in cell spinpods. The stability and reproducibility of growth makes suspension culture a simple method for introducing physiologic levels of shear stress to cells in vitro. With the availability of inexpensive, easy-to-use cell spinpods, researchers now have a simple, affordable tool to expose multiple replicates of cells to uniform low levels of fluid shear stress.

Precisely quantifying fluid mechanical forces experienced by cell suspension cultures at various operating conditions is critical to mimicking in vivo physiology and pathophysiology^33^. Specifically, the simple models of terminal velocity and maximum shear stress (above) can be generalized through the use of continuum mechanics in 3D. In doing so, precise predictions can be made regarding shear forces on cells, and how these flow forces vary throughout the volume of a rotating suspension culture^33^.

The TFM simulation of conditions within the rotating cell spinpod further highlights the nonuniform distribution of particles throughout the cross-section of the cell chamber as shown in Fig. 3. The 3D suspension velocity field is not simply azimuthal (as in simple theoretical models), having some recirculation regions. Naturally, the highest stresses on the particle phase are encountered near the vessel wall (strongest shear) but rapidly decrease to a level of about 0.5 dynes/cm^2^ in the annular region slightly inward from the wall, wherein the volume fraction of particles is highest ($\approx 30\%)$.

We demonstrated in multiple ways that exposure to fluid shear stress in rotating cell spinpods maintains viability and enhances the function of renal cells. Renal cells in rotating cell spinpods: (a) released significantly lower quantities of the NGAL, a documented marker of renal cell injury in vitro and in vivo^63–67^, (b) had significantly increased endocytic receptor activity, (c) significantly increased glucose transport, (d) had significantly increased xenobiotic efflux transporter activities, (e) had significantly greater mitochondrial membrane potential, and (f) significantly increased the production of GM-CSF and IL-6 after exposure to toxic myeloma light chains. These data illustrate that renal proximal tubular cells exposed to fluid shear stress in rotating cell spinpods remain physiologically responsive and could provide a simple model to investigate renal inflammation, fibrosis, and nephrotoxicity.

RPTEC/TERT1 cells exposed to fluid shear stress in rotating cell spinpods took up more FITC-albumin and more FITC-dextran than did cells from static cell spinpods indicating increased megalin and cubilin activity; they also transported more glucose. Upregulation of endocytic receptor activity and glucose transport by fluid shear stress has been described in a variety of renal cell models from HK-2, to rat and human primary proximal tubular cells, LLC-PK1, and conditionally immortalized human proximal tubular cells^7,8,10–12,39,68^. RPTEC/TERT1 cells exposed to fluid shear stress in rotating cell spinpods also had more xenobiotic transporter activity as demonstrated by increased efflux of GS-MF. Our results concur with those of Ross et al.^69^ who observed increased albumin uptake and increased GS-MF efflux in in RPTEC/TERT1 cells growing on a collagen-coated surface and exposed to 0.5 dynes/cm^2^ of fluid shear stress for 24 h in a chip-based microfluidic device. Compared to the other methods, cell spinpods are less expensive, simpler and easier to automate.

To further validate the nephrotoxin sensitivity of renal cells exposed to fluid shear stress in rotating cell spinpods, we exposed the cells to myeloma light chains, which are also taken up by megalin^60^. Myeloma light chains induce an assortment of renal injuries with deposition in the glomerulus inducing glomerulonephritis, megalin mediated proximal tubular uptake, including induction of transport deficits such as Fanconi system, as well as interstitial nephritis and distal tubule cast nephropathy^58,59^. Renal cells in rotating cell spinpods released significantly more GM-CSF in the presence of myeloma light chains from one donor, where as they release significantly more IL-6 in the presence of myeloma light chains from the other donor. The release of both GM-CSF and IL-6 was stimulated by exposure to myeloma light chains, which are a known nephrotoxin. Exposure to fluid shear stress in combination with myeloma light chains increased the quantity of GM-CSF and IL-6 released even further, in a myeloma light chain donor-specific manner. Renal cells release GM-CSF and IL-6 when injured or stressed^70,71^.

During rotational cell spinpod culture, addition of the nephrotoxins doxorubicin and cisplatin to the media, both reversed increased glucose and FITC albumin uptake, as well as reducing the overall glucose and FITC-albumin uptake by the renal cells. Doxorubicin and cisplatin are chemotherapeutic agents with known nephrotoxic side effects and 3D models of renal tissue have demonstrated increased sensitivity to both drugs in vitro^72^.

Gene expression analysis of renal proximal tubular cells in suspension culture has some predictable elements as well as several unexpected observations. Multiple heat shock proteins were among those genes manifesting the largest changes in gene expression, which is not surprising given their behavior in response to a wide variety of stressors. Similarly, several of the Cytoscape/ClueGo gene expression categories correlate with our functional studies that demonstrate changes in cell cycle, cellular stress, oxidation reduction process, and extracellular stimuli. Perhaps, even more telling may be the genes whose expression did not change. Cubilin provides an exemplar—the protein is so stable that there is scant RNA signal, to the point that investigators had to resort to embryonic tissue to find enough RNA signal to clone it^57^. Several expression changes in individual genes that have been observed by other groups are less significant in our analysis; this could be due to the combination of several factors, including: differences in cell type, culture method, number of replicates, timing, and the intensity of fluid shear stress applied to the cells^2,20^. Importantly, when considering future drug sensitivity studies, all of the changes in expression of drug transporters were preserved, except for a small decrease in ABCG2. Ross et al. identified the 15 genes with the top significant changes induced by exposure of RPTEC/TERT1 cells to fluid shear stress in their microfluidic device^69^. Six of the 15 genes were also significantly changed in our studies of RPTEC/TERT1 exposed to fluid shear stress in rotating cell spinpods: AKR1C1 (regulation of aldo–keto reductases), AKR1B10 (mitochondrial aldo–keto reductases with activity towards steroids and 3-keto-acyl-CoA conjugates), CYP4F11 (cytochrome P450 (CYP) enzymes), SLC44A2 (drug transporters of the organic anion transporter (OAT) family), NGFR (rapamycin-induced autophagy protects proximal tubular renal cells against proteinuric damage), and SLC43A2 (essential amino acid transporter). Taken together, our differential expression analysis should serve as a useful reference dataset against which future experiment on additional cell types and drug exposures can be compared.

Cell spinpods can be utilized in diverse configurations to answer disparate scientific questions^6,73^ (Table 2). They are amenable to the culture of mammalian cells, insect cells, and microorganisms from bacteria to fungi and viruses. Spheroids in rotating cell spinpods maintain their globe shape and can be assayed without transfer to static 2-D cultures. Renal tubular epithelial cells line the tubules of the kidney as a monolayer where they are exposed to fluid shear stress from urine. RPTEC/TERT1 cells will not grow as a spheroid without carrier beads. Even when encouraged to form spheroids by prior incubation on AggreWell™ plates (Stemcell Technologies, Vancouver, CA, USA), RPTEC/TERT1 do not hold together as a spheroid. Nor would spheroids have been a desirable model for the present studies where we wanted to expose a monolayer of renal cells to uniform fluid shear stress. However, we have observed that A549 lung carcinoma cells and HEK human embryonic kidney cells will form spheroids in rotating cell spinpods (author observation). If anaerobic cultures are desired, it is facile to replace the breathable membranes with gas impermeable membranes. Cell spinpods can be used in biofilm experiments by replacing one breathable side membrane with a suitable growth matrix. In the similar configuration, partially-filled cell spinpods can function as a miniature roller bottle, with cells exposed to alternating fluid and air environments during rotation. Cell spinpods can be injection molded in sizes from 100 µL to 100 ml or greater, reintroduce shear with a coaxial rod to mimic chronic renal and liver disease shear levels, or incorporate a bubble trap, as well as supporting biofilm studies.

One area ripe for the application of cell spinpod culture is liver and kidney toxicity- each representing major obstacles for safe drug development^2,20^. There are well-established, FDA-approved, high throughput screens for hepatotoxicity, but scant if any for nephrotoxicity^74,75^. Modeling the full anatomical and functional complexity of a kidney is not a cost-effective approach for high throughput screening. Proximal tubule cells are a logical selection for in vitro screening as they take up and metabolize the majority of renally-filtered drugs and are the most frequent renal cell to show the toxic effects of drugs. Shear stress can maintain the differentiation of this key cellular target in vitro. Proximal tubule kidney cells cultured in suspension culture display differentiated features, such as expression of megalin, cubilin, and microvilli not seen in most static 2-D cultures^5,10,37,76^. Renal cells cultured in rotating cell spinpods under shear flow conditions provide a facile and informative model for evaluating nephrotoxicity^6^.

Cell spinpods provide a new, inexpensive, easy-to-use, simply automated tool for diverse physiological, pathological, and toxicity applications.

## Data availability

All sequencing data is available through NASA GeneLab repository.

## Supplementary Information

Supplementary Figure S1.

Supplementary Figure S2.

Supplementary Information 1.

Supplementary Information 2.

Supplementary Information 3.

Supplementary Information 4.

Supplementary Information 5.

Supplementary Information 6.

Supplementary Information 7.

Supplementary Information 8.

Supplementary Information 9.

Supplementary Table S2.

Supplementary Video S1.

Supplementary Video S2.

Supplementary Legends.
