# Author Correction: Cell spinpods are a simple inexpensive suspension culture device to deliver fluid shear stress to renal proximal tubular cells

**DOI:** 10.1038/s41598-021-02538-y

**Published:** 2021-11-24

**Authors:** Timothy G. Hammond, Corey Nislow, Ivan C. Christov, Vecihi Batuman, Pranay P. Nagrani, Marjan Barazandeh, Rohit Upadhyay, Guri Giaever, Patricia L. Allen, Michael Armbruster, Allen Raymond, Holly H. Birdsall

**Affiliations:** 1 Cell Spinpod LLC, Chapel Hill, NC 27516 USA; 2 Nephrology Division, Department of Internal Medicine, Duke University School of Medicine, Durham, NC 27705 USA; 3 Space Policy Institute, Elliott School of International Affairs, George Washington University, Washington, DC 20052 USA; 4 Nephrology Section, John W. Deming Department of Medicine, Tulane University School of Medicine, New Orleans, LA 70112 USA; 5 Nephrology Section, Medicine Service Line, Durham VA Health Care System, Building 15, Room 210, 508 Fulton Street, Durham, NC 27705 USA; 6 Faculty of Pharmaceutical Sciences, The University of British Columbia, Vancouver, BC V6T 1Z3 Canada; 7 School of Mechanical Engineering, Purdue University, West Lafayette, IN 47907 USA; 8 Incept 3D, San Diego, CA 92121 USA; 9 Rite Tech Industries Inc., Trinity, FL 34655 USA; 10 Departments of Otorhinolaryngology, Immunology, and Psychiatry, Baylor College of Medicine, Houston, TX 77030 USA; 11 Otolaryngology Section, Surgery Service Line, Durham VA Health Care System, Building 15, Room 210, 508 Fulton Street, Durham, NC 27705 USA

Correction to: *Scientific Reports* 10.1038/s41598-021-00304-8, published online 29 October 2021

The original version of this Article contained errors in the key for Figures 7, 8 and 9 where the solid red square “rotating” was incorrectly given as “static”, and the striped square “static” was incorrectly given as “rotating”. The original Figures 7, 8 and 9 and accompanying legends appear below.

**Figure 7:**
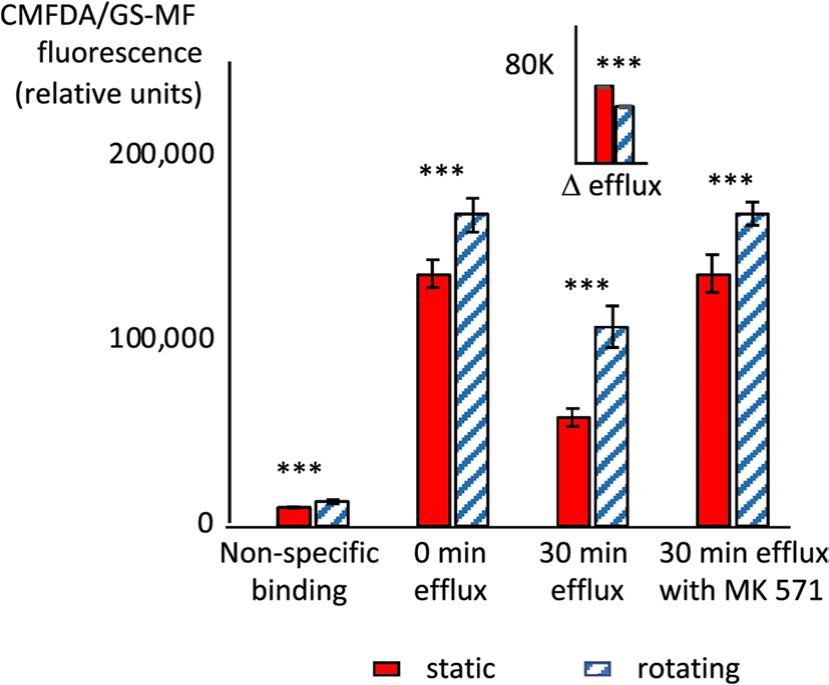
Effect of rotation in cell spinpods on the activity of xenobiotic efflux transporters. RPTEC/TERT1 cells on Cytodex carrier beads were cultured in cell spinpods under rotating () or static conditions () for 48 h and harvested. One aliquot of cells was chilled and cold CMFDA was added before immediate analysis to measure non-specific binding. The remaining cells were incubated with CMFDA for 40 min at 37 °C, washed, and the CMFDA/GM-SF was allowed to efflux in the presence and absence of the inhibitor MK571. The quantity of CMFDA/GS-MF in the cells was measured by flow cytometry immediately after washing (0 min efflux) and after 30 min of efflux at 37 °C (30 min efflux and 30 min efflux with MK 571). Asterisks indicate where differences in the distributions between groups were statistically significant, e.g. *p* < 0.05, by one-tailed Mann–Whitney *U* test. Non-specific binding was significantly higher in cells from rotating cell spinpods (*p* = 0.00015, *W* = 2, *n*_1_ = *n*_2_ = 5). CMFDA/GS-MF remaining in cells from rotating cell spinpods was significantly lower at time zero (*W* = 4, *n*_1_ = *n*_2_ = 6, *p* = 0.013), after 30 min of efflux (*p* = 0.00035, *W* = 0, *n*_1_ = *n*_2_ = 5), and after 30 min of efflux in the presence of MK571 (*p* = 0.065, W = 6, *n*_1_ = *n*_2_ = 6). The inset shows the difference in CMFDA/GM-SF signal between time 0 and after 30 min of efflux. The loss of CMFDA/GM-SF from cells in rotating cell spinpods was significantly larger (*p* = 0.008, *W* = 24, *n*_1_ = *n*_2_ = 5).

**Figure 8:**
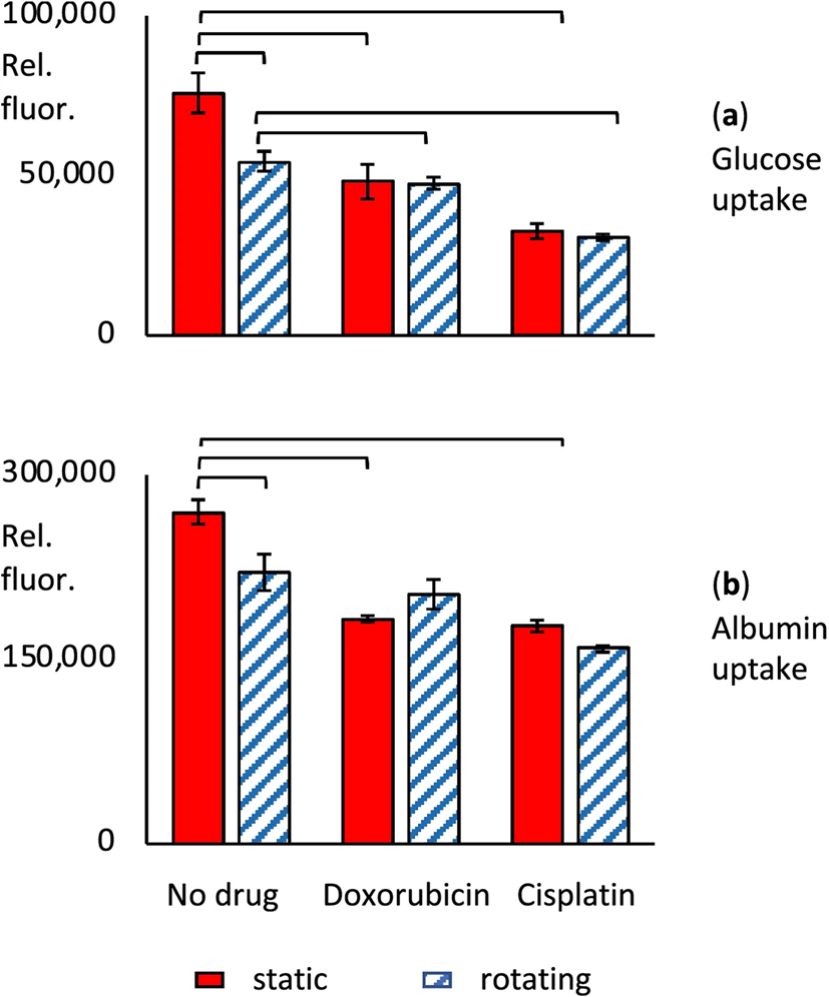
Effect of rotation in cell spinpods on effect of chemotherapeutic agents. RPTEC/hTERT cells on carrier beads were cultured in cell spinpods under rotating conditions () or static conditions () for two days in the presence of 5 µM doxorubicin, 100 µM cisplatin, or no drug control. Panel (**a**) shows the uptake of glucose, measured with the fluorescent substrate 2-NDBG. Panel (**b**) shows the uptake of FITC-albumin. Cells from all samples were trypsinized off the carrier beads before analysis by flow cytometry. Data is presented as mean fluorescence ± SEM of six replicates (except for 5 replicates with static cisplatin), in relative fluorescence units. Brackets mark where differences in the distributions between groups were statistically significant, e.g. *p* < 0.05, by one-tailed Mann–Whitney *U* test.

**Figure 9:**
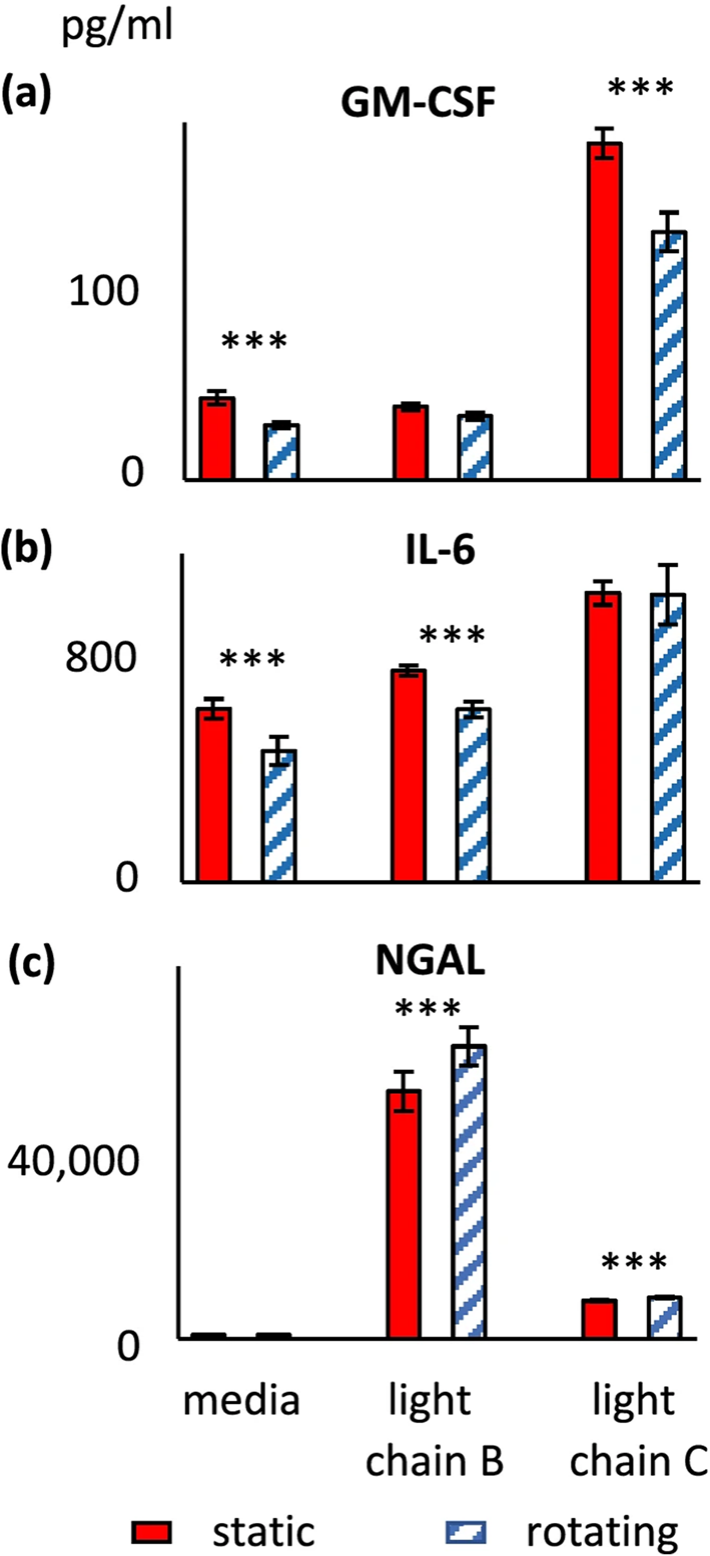
Effect of rotation in cell spinpods on cytokine release from renal cells. RPTEC/TERT1 cells on Cytodex carrier beads were cultured in cell spinpods under rotating conditions () or static conditions () in the presence of myeloma light chains from donor B, myeloma light chains from donor C, or media. After 48 h, supernatants were harvested for assay of cytokines. Bars presented mean concentration (pg/mL) of GM-CSF, panel (**a**), IL-6, panel (**b**), and NGAL panel (**c**), in the cell supernatant; error bars are ± SEM. Asterisks indicate where differences in the distributions between rotating and static groups were statistically significant, e.g. *p* < 0.05, by one-tailed Mann–Whitney *U* test. Rotation induced significantly greater quantities of GM-CSF in the media control (*p* = 0.004, *W* = 39, *n*_1_ = 7, *n*_2_ = 6) and after stimulation with myeloma light chains from donor C (*p* = 0.005, *W* = 33, *n*_1_ = 7, *n*_2_ = 5). Rotation induced significantly greater quantities of IL-6 after culture in media alone (*p* = 0.036, *W* = 34, *n*_1_ = 7, *n*_2_ = 6) and with myeloma light chains from donor B (*p* = 0.001, *W* = 41, *n*_1_ = 7, *n*_2_ = 6). Rotation significantly reduced the quantities of NGAL released after stimulation with myeloma light chains from donor C (*p* = 0.024, *W* = 5, *n*_1_ = 7, *n*_2_ = 5) and myeloma light chains from donor B (Fig. 8c, *p* = 0.05, *W* = 9, *n*_1_ = 7, *n*_2_ = 5).

The original Article has been corrected.

