# Supplementary material for: Cell spinpods are a simple inexpensive suspension culture device to deliver fluid shear stress to renal proximal tubular cells
Source: Sci Rep. 2021 Oct 29;11:21296. doi: 10.1038/s41598-021-00304-8 (PMC8556299; doi:10.1038/s41598-021-00304-8)

Supplemental Figure S1 Panel (a)

State 1 = 0 hours
State 2 = 3 hours

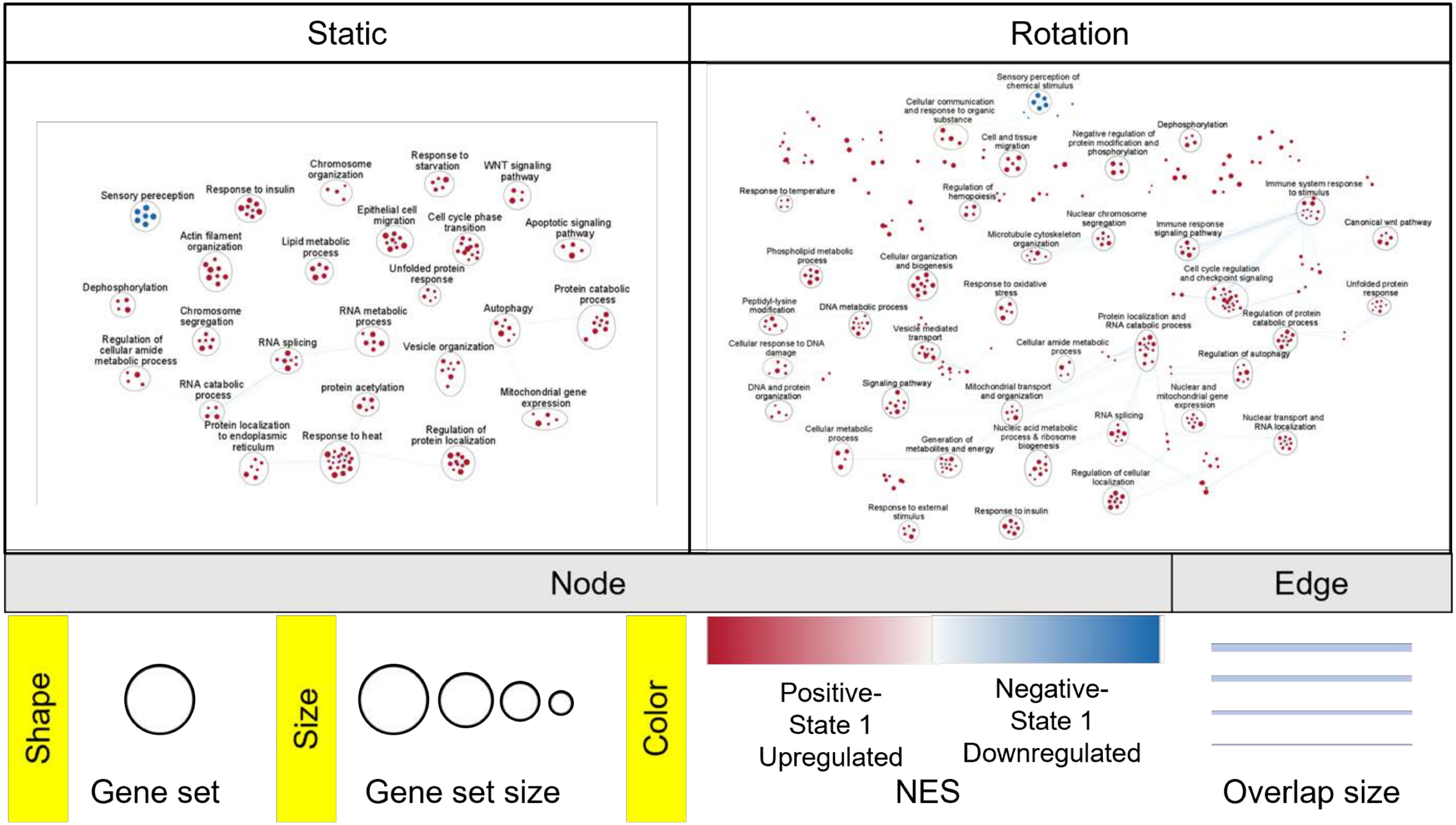

Supplemental
Figure S1 Panel (b)

State 1 = 0 hours
State 2 = 24 hours

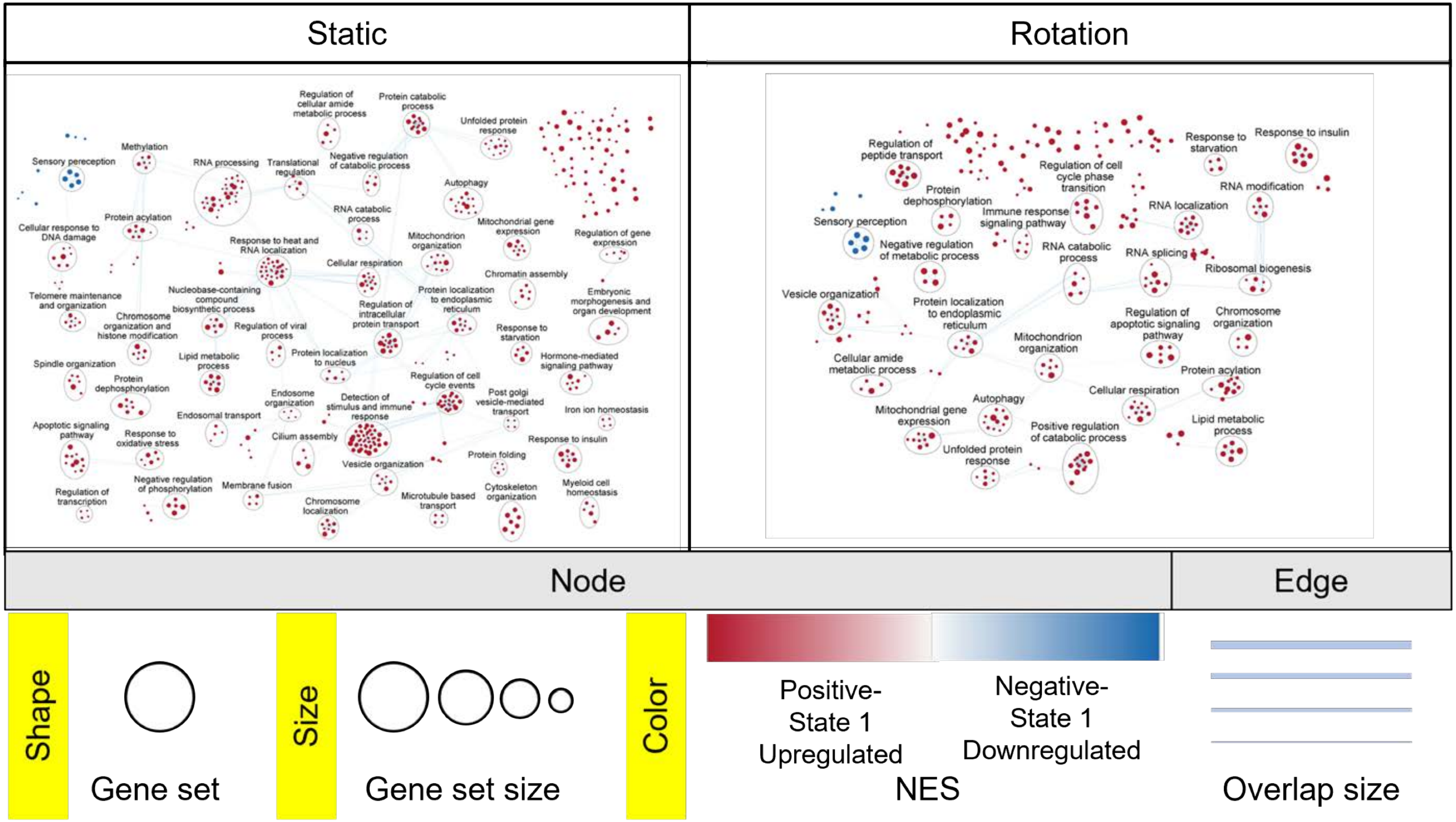

Supplemental
Figure S1 Panel (c)

State 1 = 0 hours
State 2 = 72 hours

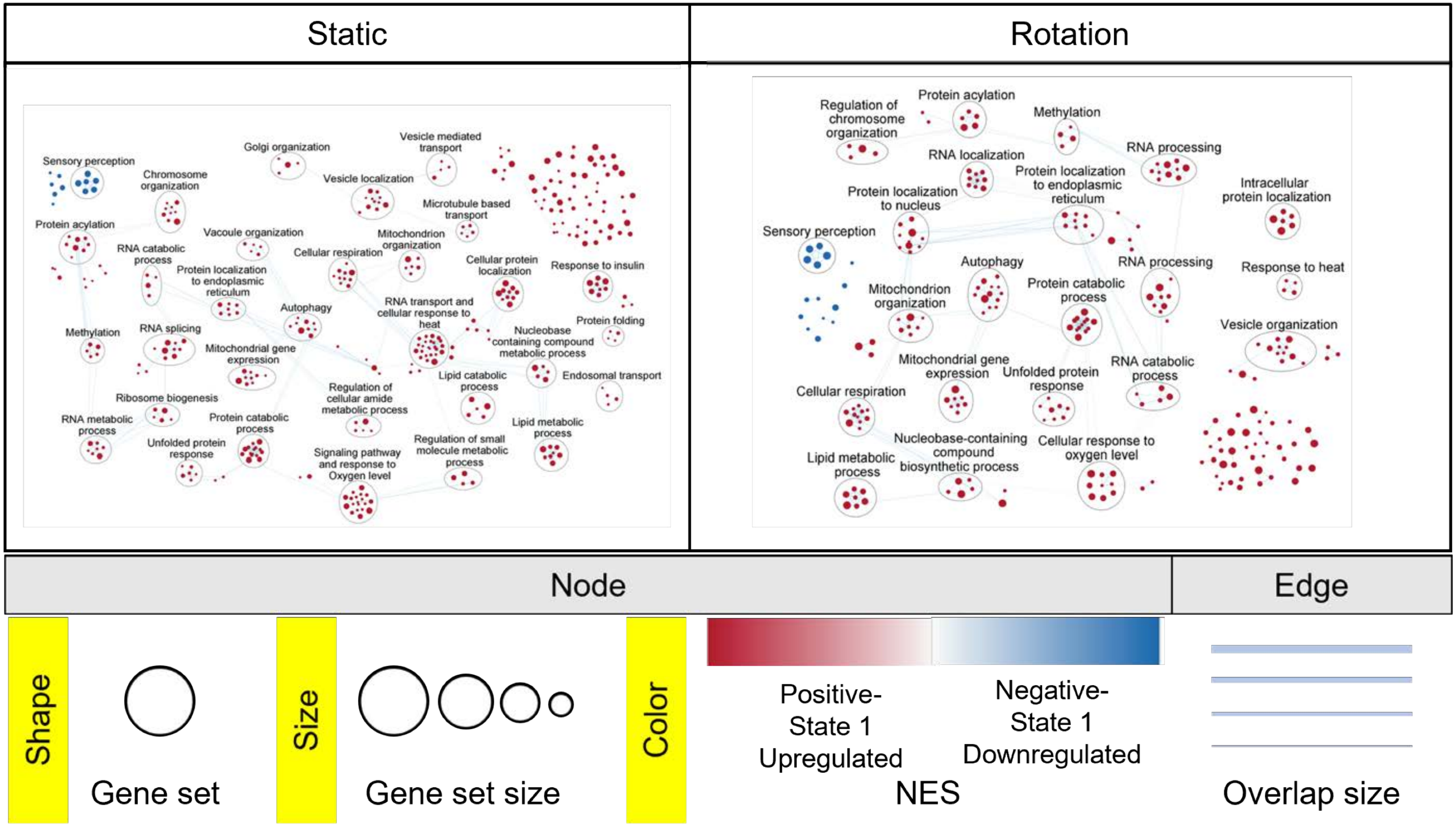

Supplement: Supplementary file 1 — Supplementary Figure S1. [file 41598_2021_304_MOESM1_ESM.pdf]
