# Supplementary figures and images for: Cell spinpods are a simple inexpensive suspension culture device to deliver fluid shear stress to renal proximal tubular cells
Source: Sci Rep. 2021 Oct 29;11:21296. doi: 10.1038/s41598-021-00304-8 (PMC8556299; doi:10.1038/s41598-021-00304-8)

Supplemental  
Figure S2

(a) 3 hours

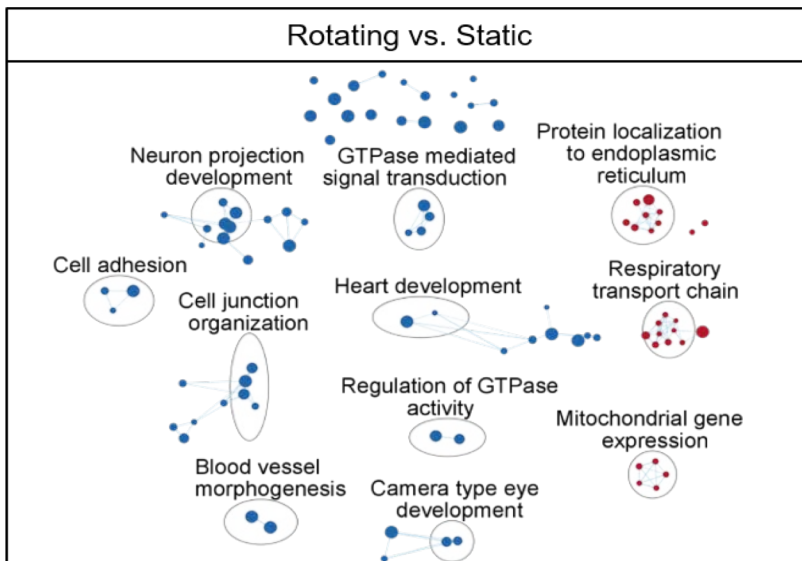

(b) 24 hours

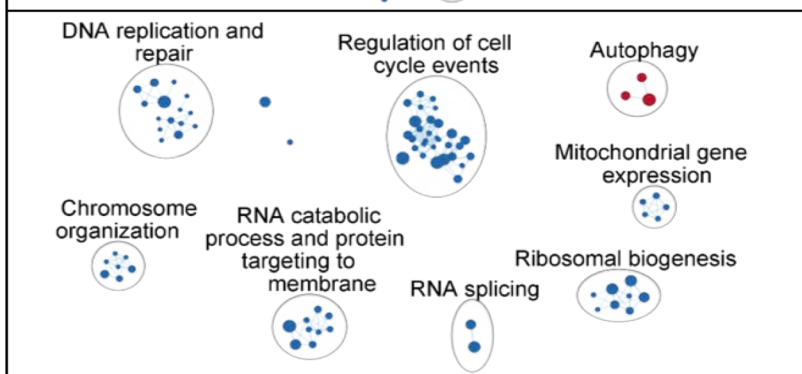

(c) 72 hours

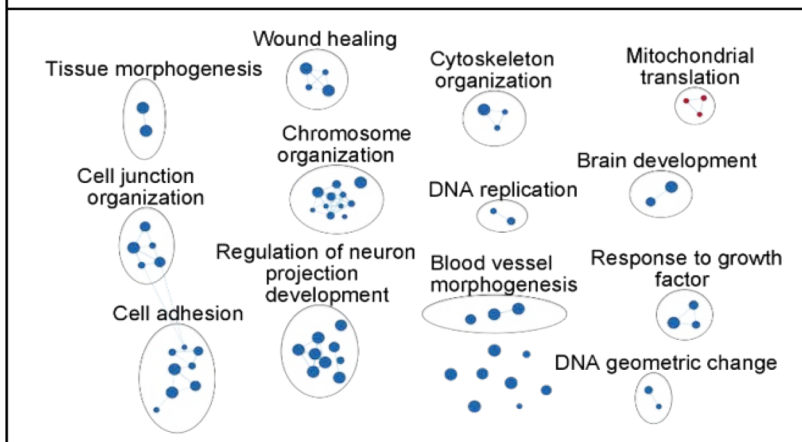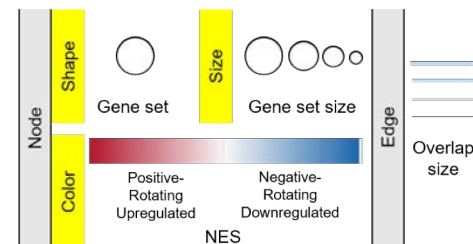

Supplement: Supplementary file 2 — Supplementary Figure S2. [file 41598_2021_304_MOESM2_ESM.pdf]
