# Supplementary material for: Cell spinpods are a simple inexpensive suspension culture device to deliver fluid shear stress to renal proximal tubular cells
Source: Sci Rep. 2021 Oct 29;11:21296. doi: 10.1038/s41598-021-00304-8 (PMC8556299; doi:10.1038/s41598-021-00304-8)
Supplement: Supplementary file 15 — Supplementary Legends. [file 41598_2021_304_MOESM15_ESM.docx]

**Supplemental Data**

**Video 1: Beads in rotating cell spinpod.** The video shows the path of Cytodex beads, stained with blue dye in a cell spinpod that is initially at rest and then begins to spin as the bottle roller is accelerated in step-wise increments. Video was prepared by the authors TH and HB.

**Video 2: Loading of cell spinpods.** The video shows the loading process for cell spinpods using gravity feed. The media is introduced through an infusion set using an 18-gauge needle. A 27-gauge needle in the upper port allows the evacuation of air as the cell spinpod fills. Video was prepared by the authors TH and HB.

**Supplemental Figure S1. Enrichment maps of RPTEC/TERT1 cells in 0 time versus 3-, 24-, and 72-hour time points in rotating and static conditions.** Enrichment maps are generated in Cytoscape v. 3.8.2 using the EnrichmentMap application v. 3.3.2 (https://Cytoscape.org). Red and blue nodes represent state 1 versus state 2 upregulated and downregulated gene sets, respectively. For clarity, only nodes with FDR q-value = 0 and edge similarity cuttoff > 0.5 are represented. The size of the nodes is proportional to number of genes involved in each pathway. The edges between nodes represents the mutual overlap between the nodes and the edge width reflects the number of genes that overlap between the two nodes. Overlapping gene sets with the top NES values (> 3.5 or < -3.5) are clustered together using AutoAnnotate application v. 1.3.4 in Cytoscape (https://Cytoscape.org). Only clusters with > 3 members are circled and labelled. The pathways, genes and variables associated with each node in the enrichment map can be found in Supplemental Table S2.

**Supplemental Figure S2. Enrichment maps of RPTEC/TERT1 cells in rotation versus static conditions at 3-, 24-, and 72-hour time points.** Enrichment maps are generated in Cytoscape v. 3.8.2 using the EnrichmentMap application v. 3.3.2 (https://Cytoscape.org). Red and blue nodes represent state 1 versus state 2 upregulated and downregulated gene sets, respectively. For clarity, only nodes with FDR q-value = 0 and edge similarity cuttoff > 0.5 are represented. The size of the nodes is proportional to number of genes involved in each pathway. The edges between nodes represents the mutual overlap between the nodes and the edge width reflects the number of genes that overlap between the two nodes. Overlapping gene sets with the top NES values (> 3.5 or < -3.5) are clustered together using AutoAnnotate application v. 1.3.4 in Cytoscape (https://Cytoscape.org). Only clusters with > 3 members are circled and labelled. The pathways, genes and variables associated with each node in the enrichment map can be found in Supplemental Table S2.

**Supplemental Table S1: Differentially expressed genes and their significance based on the FDR q-value in RPTEC/TERT1 cells, rotation versus static conditions at 3-, 24-, and 72-hour time points.** FPKM (fragments per kilobase of exon model per million reads mapped) is a normalized estimation of gene expression based on RNA-seq data. q-values are FDR adjusted p-values.

**Supplemental Table S2. Node tables of enrichment maps represented in supplemental figures S1 and S2.** Each table contains pathways, genes and variables associated with each node in the enrichment maps in supplemental figures S1 and S2.
